# Promoting the development and approval of new traditional Chinese medicines in China: a pooled analysis of data from 2013 to 2024

**DOI:** 10.3389/fmed.2025.1559703

**Published:** 2025-06-16

**Authors:** Xiaohong Long, Jie Zhang, Li Yang

**Affiliations:** School of Business Administration, Shenyang Pharmaceutical University, Shenyang, China

**Keywords:** traditional Chinese medicine, evaluation and registration evidence, clinical trials, development and approval reform, China

## Abstract

The reform of the development and approval system for traditional Chinese medicines (TCMs) has been in progress for several years. This reform has restructured the registration classifications and established a distinctive evaluation and registration evidence system for TCMs. This study compiled comprehensive data on all new TCMs approved in China from January 2013 to November 2024, analyzing drug characteristics and the changes in development and review timelines before and after the reform. The focus was particularly on the evaluation and registration evidence requirements, clinical development pathways, and the application of pivotal clinical and real-world evidence in supporting TCM approvals. Between 2013 and 2024, 77 new TCMs were approved for marketing. Post-reform, there has been a gradual increase in the number of new TCMs, with a notably significant rise in ancient classic formulas. Following 2020, the establishment of the “three-in-one” evaluation and registration evidence system was implemented to accommodate the distinct characteristics of TCMs. This system revealed significant discrepancies in the registration classifications for new TCMs, particularly concerning TCM theory, human use experience, and clinical trials. These discrepancies have led to varied clinical development pathways. Importantly, the findings suggest that the marketing approval of new TCMs is no longer solely reliant on Randomized Controlled Trials. Instead, TCM theories and empirical human use experience constitute critical evidence for TCM approval. Additionally, evidence derived from real-world studies has become instrumental in supporting the marketing of TCMs. Although the review time for TCMs has significantly decreased after the reform, the overall development time has increased. Simultaneously, this article proposed specific recommendations to address the array of challenges encountered by new TCMs in the realms of development and approval.

## 1 Introduction

Recently, China has focused significant attention on the development and approval of new traditional Chinese medicines (TCMs) and has actively introduced various policies, regulations, and relevant technical guidelines to encourage and support this sector. In 2016, the Law of the People’s Republic of China on Traditional Chinese Medicine issued by the National People’s Congress and the Outline of the Strategic Plan for the Development of Traditional Chinese Medicine (2016–2030) (1, 2) issued by the State Council, explicitly proposed that efforts should be made to promote the innovation of TCMs. These two documents underscored the importance of both preserving TCM heritage and fostering innovation as fundamental strategies for the TCM development and approval (3). In 2019, the State Council released the Opinions on Promoting the Passing Down, Innovation, and Development of Traditional Chinese Medicines, which further highlighted the need to enhance mechanisms for TCM inheritance and innovation (4). Furthermore, the document advocated for the implementation of a “three-in-one” evaluation and registration evidence system that integrated TCM theory, human use experience, and clinical trials to comprehensively evaluate the safety, effectiveness, and quality controllability of TCMs. In 2020, the revised Provisions for Drug Registration released by the National Medical Products Administration (NMPA) which was the indicator of the official launch of the reform of the development and approval of TCMs, significantly adjusted the registration classification of TCMs and emphasized that TCM development and approval should be guided by clinical value. Concurrently, four expedited programs were established to accelerate the development and approval of drugs for marketing: breakthrough therapy designation program (BTD), conditional approval (CA), priority review (PR), and special review and approval procedure (SRAP) (5).In the same year of 2020, the NMPA articulated in the Guiding Principles for Real World Evidence to Support Drug Research and Development and Evaluation (Trial) that real-world research can be utilized as a methodology for the clinical development and assessment of TCMs with extensive clinical application histories, thereby providing a novel developmental pathway and foundation for evaluating the inheritance and innovation of TCMs (6). In 2023, the NMPA promulgated Specialized Management Regulations for the Registration of TCMs, clarifying the rational application of human use experience in the development and approval of TCMs (7). It also further improved the TCM evaluation and registration evidence system that combines TCM theory, human use experience, and clinical trials through necessary technical requirements. In addition, a series of technical guidelines, including the Clinical Research and Development Guidelines for TCM Compound Preparations based on Human Experience (Trial), and the Communication and Exchange Guidelines based on the “Three in One” Registration and Evaluation Evidence System (Trial), have also been implemented simultaneously to guide the development and approval of TCMs (8, 9). Supplementary Figure 1 show other highly representative policies, regulations, and relevant technical guidelines on TCMs. With the driver of a series of regulations and policies, the number of new TCMs in China has shown a gradually increasing trend in recent years. While some scholars have investigated the development and approval of new TCMs (10–12), most of these studies have predominantly focused on the period preceding 2020. Consequently, there is a notable paucity of research concerning newly marketed TCMs following the reform of the development and approval system post-2020. This article aimed to provide a comprehensive overview of the development trajectories and approval trends of new TCMs in China from 2013 to 2024. It examined the evaluation and registration evidence requirements, clinical development pathways, and the application of pivotal clinical and real-world evidence in supporting TCM approvals. Additionally, it analyzed the characteristics and patterns of development and review time for TCMs under the revised registration and evaluation evidence system implemented after 2020. This study represented the first attempt to utilize approval data to assess the impact of the reform on the development and approval of new TCMs.

## 2 Materials and methods

### 2.1 Study design and sample

This study investigated the new TCMs that received approval from the NMPA of China between January 2013 and November 2024. In this research, each new TCM with its first approved indication was treated as a single entity for analysis, referred to as an “new TCM item.” This approach resulted in 77 new TCMs.

### 2.2 Data source and extraction

The criteria for the inclusion of new TCMs were delineated and categorized in accordance with the stipulations set forth in the Provisions for Drug Registration. During the period from 2013 to 2024, two iterations of these criteria were promulgated and enacted in China: the 2007 version and the 2020 version, respectively. The 2007 edition of the Provisions for Drug Registration identified nine classifications of TCMs, with classes 1 through 6 designated as new drugs. Conversely, the 2020 Provisions for Drug Registration reconsolidated TCMs into four major classifications, with classes 1 to 3 identified as new drugs (13, 14). Detailed changes in the registration classification of TCMs were presented in Supplementary Table 1.

**FIGURE 1:**
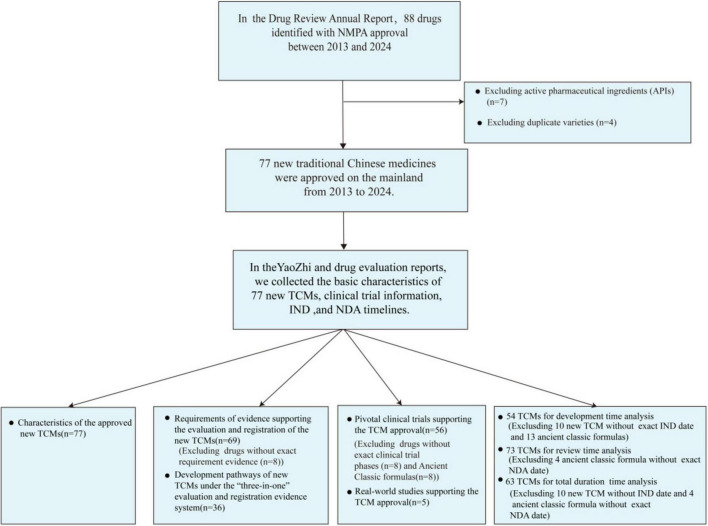
Flow diagram of drug selection.

We conducted a comprehensive search for TCMs that have received marketing approval between January 2013 and November 2024, utilizing the Drug Review Annual Report issued by the Center for Drug Evaluation (CDE) of the NMPA and the widely recognized business database YAOZHI China (15, 16). For the period from 2013 to 2020, we included TCMs classified under categories 1 to 6 in our statistical analysis. From 2021 to 2024, our analysis was restricted to TCMs classified under categories 1 to 3. Active pharmaceutical ingredients (APIs) were excluded from this study due to their indirect availability for patient use and distinct regulatory requirements. In accordance with the 2020 registration version, which classifies ancient classical prescriptions under category 3, China allows multiple companies to seek marketing approval for identical formulations. For this analysis, only the first approved formulation based on ancient classical prescriptions was considered. The detailed search methodology was illustrated in Figure 1.

In the drug evaluation reports published by the CDE, we extracted information on the type of approval, registration class, clinical trial phase, and pivotal clinical trial characteristics which encompassed trial design, blinding, control settings, and indicators for evaluating clinical efficacy for each drug. In the YAOZHI database of China, we collected various key data about the World Health Organization Anatomical Therapeutic Chemical (ATC) classification of TCM, the date of approval of the investigational new drug (IND), the date of the receipt of the new drug application (NDA) and the authorization of the first marketing approval in China, the status of expedited program designations, and the origin of the TCMs (domestic or imported). Furthermore, we gathered the data of real-world studies supporting the marketing approval of TCMs by reviewing publicly accessible literature from PubMed and Web of Science. This information was further augmented with reports available on the related company’s official website. The criteria for identifying rare diseases were derived from the catalog of rare diseases, which serves as a reference issued by the National Health Commission (NHC). The therapeutic area was categorized according to the ATC Classification System, and the origin of the TCMs was classified as domestic or imported depending on the manufacturer’s location within or outside of mainland China. Explanations of all the relevant terms mentioned in this article were listed in Supplementary Table 2.

### 2.3 Statistical analysis

To examine the progress in the development and approval of the TCMs following the reform, we conducted an analysis of data segmented into two distinct time periods based on the year of approval: 2013–2020 (pre-2020) and 2021–2024 (post-2020).

The numerical data were expressed as medians and quartiles. Descriptive statistics were calculated for the number and percentage of TCMs in each category. The distributional characteristics of the categorical variables were compared using Fisher’s exact test (17). Clinical development time was calculated as the number of months that elapsed from the IND approval date to the NDA submission date. Review time was defined as the months from the submission date of the NDA to the market authorization date. The total duration was defined as the period between the IND approval date and the marketing authorization date (18, 19). In this study, non-parametric Mann–Whitney U tests were employed to ascertain the existence of differences in development time, review time, and total duration across specified periods (2013–2020, 2021–2024) (20). The statistical analyses were performed using SPSS 27.0 and GraphPad Prism 9.0. A two-tailed *p*-value < 0.05 was considered statistically significant. R4.4.2 was used to draw the clinical development roadmap.

## 3 Results

### 3.1 Characteristics of the approved new TCMs

Between 2013 and 2024, the NMPA granted marketing approvals for a total of 77 new TCMs (Supplementary Table 3). From 2013 to 2017, the number of new TCMs approved for marketing declined annually, with only 1 (1.3%) approved in 2017. The number of new TCMs approved remained relatively steady at around 2–3 yearly in 2018–2020. Following the year 2020, the number of new TCMs has increased annually, with the number of launches reaching a peak in recent years by 2021, with 12 (15.6%) new medicines approved for marketing.

**FIGURE 2:**
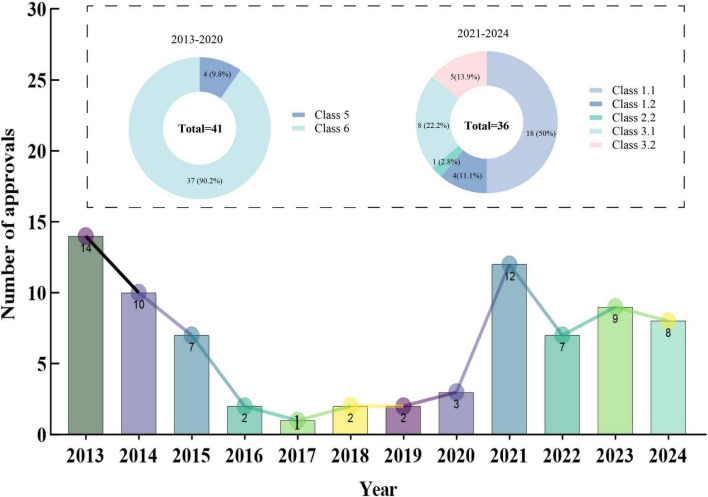
Number of new TCMs approved for marketing from 2013 to 2024.

In terms of registration categories, TCMs approved for marketing between 2013 and 2020 were predominantly concentrated in class 5 and class 6, accounting for 9.8% and 90.2% of approvals, respectively. In the period of post-2020, class 1.1 (TCM compound preparations) emerged as the predominant category, constituting 50% (*n* = 18) of approvals. This was followed by class 3 (ancient classic prescriptions), which represented 36.1% (*n* = 13), and class 2 (improved new medicines), comprising 2.8% (*n* = 1) of the approvals (see Figure 2).

When investigating the TCM type, we found that new TCMs have two types: single-prescription of TCMs and compound preparations of TCMs. Compound preparations of TCMs refer to preparations made with multiple prepared slices/decoction pieces and extracts based on TCM theories. Single-prescription of TCMs are extracts obtained from a single plant, animal, or mineral materials and their preparations. Between 2013 and 2024, the approvals of new TCMs in China were predominantly characterized by compound preparations, which constituted 88.3% of the approvals compared to 11.7% for single-prescription. Notably, there has been a recent increase in the approval proportion of single-prescription, rising from 9.8% to 13.9%. Furthermore, an analysis of origin revealed that domestic organizations were responsible for the development of 98.7% of the newly approved TCMs in China, while a mere 1.3% originated from international sources.

The leading therapeutic classes for these new TCMs included the respiratory system (21, 27.3%), genitourinary system (13, 16.9%), alimentary tract and metabolism (13, 16.9%), and nervous system (12, 15.5%). The representative TCMs for the respiratory system encompass Qingfei Paidu Granules, Huashi Baidu Granules, and Xuanfei Baidu Granules, which were utilized in the management of severe acute respiratory syndrome induced by the COVID-19 infection. In the context of genitourinary treatment, the advancement of new TCMs has predominantly concentrated on gynecological applications. For instance, Wenjing Tang Granules which serves as a notable example of a classic gynecological formula, have been officially and widely recognized in China. In the realm of digestive system therapeutics, Erdong Tang Granules, Yiguan Jian Granules, and Qirui Weishu Capsules are representative examples. For the neurological system, representative TCMs include Depressant Capsules and Ginseng and Keranium Kidney Tonic Capsules. In addition, other therapeutic areas, such as the musculoskeletal and sensory systems, demonstrated a limited approval number of new TCMs in recent years, as detailed in Table 1.

**TABLE 1:** The characteristics of approved new TCMs, 2013–2024.

	No. (%)			
	Total (*n* = 77)	Pre-2020 (*n* = 41)	Post-2020 (*n* = 36)	*P*-value^c^
TCM type				0.726
TCM single preparation	9 (11.7)	4 (9.8)	5 (13.9)	
TCM compound preparation	68 (88.3)	37 (90.2)	31 (86.1)	
Origin				0.468
Domestic	76 (98.7)	41 (100)	35 (97.2)	
Imported	1 (1.3)	0 (0)	1 (2.8)	
ATC category^a^				0.399
A	13 (16.9)	6 (14.6)	7 (19.4)	
B	4 (5.2)	4 (9.8)	0 (0)	
C	2 (2.6)	2 (4.9)	0 (0)	
D	4 (5.2)	3 (7.3)	1 (2.8)	
G	13 (16.9)	8 (19.5)	5 (13.9)	
L	1 (1.3)	0 (0)	1 (2.8)	
M	6 (7.8)	3 (7.3)	3 (8.3)	
N	12 (15.5)	6 (14.6)	6 (16.7)	
R	21 (27.3)	9 (22)	12 (33.3)	
S	1 (1.3)	0 (0)	1 (2.8)	
**Innovativeness**				< 0.001
First in class	1 (1.3)	0 (0)	1 (2.8)	
Advance in class	63 (81.8)	41 (100)	22 (61.1)	
Ancient classical formulas	13 (16.9)	0 (0)	13 (36.1)	
Rare disease drug				NA
Yes	0 (0)	0 (0)	0 (0)	
No	79 (100)	41 (100)	36 (100)	
Expedited pathway^b^				0.111
Only PR	2 (2.6)	1 (2.4)	1 (2.8)	
Only CA	1 (1.3)	0 (0)	1 (2.8)	
Only BTD	0 (0)	0 (0)	0 (0)	
Only SRAP	3 (3.9)	0 (0)	3 (8.3)	
PR and CA	1 (1.3)	0 (0)	1 (2.8)	
None	70 (90.9)	40 (97.6)	30 (83.3)	

^a^ATC category: A, Alimentary tract and metabolism; B, Blood and blood-forming organs; C, Cardiovascular system; D, Dermatological; G, Genital urinary system and sex hormones; L, Antineoplastic and immunomodulating agents; M, Musculo-skeletal system; N, Nervous system; R, Respiratory system; S, Sensory organs.

^b^PR, Priority Review; CA, Conditional Approval; SRAP, Special Review and Approval Procedure; BTD, Breakthrough Therapy Designation.

^c^*P*-values were calculated based on Fisher’s exact test. NA, not available.

Regarding innovativeness, new TCMs that have been approved for marketing predominantly concentrated on advanced-in-class innovation. Notably, new TCMs have made significant advancements in anticancer therapy, as illustrated by the conditional approval of the Epimedium soft capsule in 2022, which has demonstrated efficacy in the treatment of hepatocellular carcinoma. Furthermore, this medication currently stands as the only original new drug within the global first-in-class category of TCM.

7 out of 77 TCMs, representing 9.1%, were approved for marketing through expedited programs. The number and types of TCMs receiving expedited program designations increased significantly in the post-2020 period compared to the pre-2020 period (16.7% vs. 2.4%). Of these, three out of the seven new TCMs (42.9%) were approved in 2021 on an emergency basis through the SRAP as primary treatments for COVID-19. The remaining four TCMs include two approved solely under PR, one under CA, and one under both CA and PR. Notably, none of the newly approved TCMs in China were designated as BTD or as medicines for rare diseases, as detailed in Table 1 and Supplementary Table 4.

### 3.2 Requirements of evidence supporting the evaluation and registration of the new TCMs

In 2020, the NMPA established the “Registration Classifications and Requirements for Application Dossiers of Traditional Chinese Medicines (TCMs) (21),” delineating specific criteria for the submission of application materials related to TCM theory, human use experience, and clinical trials. TCM theory serves as the foundational basis for developing scientific hypotheses regarding new TCMs It offers logical explanations for the efficacy and intended demographic of prescriptions, drawing from ancient medical texts, contemporary theoretical research on TCM, or expert medical discourse (22). The humans use experience is the core of the inheritance and innovation of TCMs, encompassing the comprehension and synthesis of the target population, dosage, efficacy characteristics, and clinical benefits of TCMs as observed in clinical practice (23). In this study, based on the origin of the formulas, newly approved TCMs were classified into four categories concerning clinical experience in human application: ancient classical formulas, clinical experience formulas, medical institution TCM preparations, and pharmacology-based screening of TCMs. Clinical trials, acknowledged as a standard approach for assessing the efficacy and safety of medications (24), provide additional validation of their clinical efficacy grounded in TCM theory and human use experience. Our study categorized clinical trials into five distinct types according to the clinical developmental pathway of TCMs. These categories include Phase I, II, and III trials; Phase II and III trials; Phase III trials; real-world studies; and clinical waivers. We explained the relationship between the three specifically in Supplementary Figure 2.

After excluding eight new TCMs that lacked clinical trial data, we incorporated 69 new TCMs into our analysis (refer to Supplementary Table 6). Our findings revealed that none of the 33 new TCMs approved prior to 2020 explicitly adhered to TCM theoretical guidance. Conversely, the proportion of new TCMs approved post-2020 that were supported by TCM theories increased significantly, reaching 88.9%. With regard to registration classes, a notable divergence was observed in the presence of TCM theoretical support among new TCMs across different registration categories in the post-2020 period (*P* = 0.008). Specifically, 94.4% of class 1.1 TCMs, 25% of class 1.2 TCMs, and all class 2.2 and class 3 TCMs were supported by TCM theories. The data indicated that new TCMs approved for marketing were predominantly composed of clinically experienced formulas both pre-2020 (54.5%) and post-2020 (44.4%). However, the prevalence of ancient classical formulas increased significantly post-2020 (36.1% compared to 18.2%). From the perspective of registration classes, the data suggested that prior to 2020, all class 5 TCMs were derived from pharmacology-based screening and lacked human use experience. In contrast, 62.1% of the class 6 TCMs were derived from clinical experience formulas. Following the year 2020, a significant variation in human use experience was noted across different registration classes (*p* < 0.001). Specifically, 83.3% of class 1.1 TCMs were derived from clinical experience formulas. In contrast, all class 3 TCMs originated from ancient classical formulas with a long history of human use. Conversely, class 1.2 preparations were exclusively derived from pharmacology-based screening of TCMs, lacking any prior human use experience. Our data indicated that the majority of new TCMs have progressed through various stages of clinical trials, with a considerable number reaching phases II and III. Between 2013 and 2020, all 33 new TCMs completed comprehensive premarket clinical trials (Phases I, II, III) or at least Phase II and III trials. In the period following 2020, a significant methodological shift in clinical trials was observed (*p* < 0.001). The data reveal that 16 out of 18 class 1.1 TCMs (88.8%) underwent phase II and III clinical trials, while all four class 1.2 TCMs completed phases I, II, and III clinical trials. Significantly, a new TCM in an enhanced dosage form has progressed to the phase III clinical trial stage. Beyond conventional clinical trial methodologies, all five new TCMs classified as class 3.2 were assessed through real-world studies. Additionally, all eight TCMs classified as class 3.1 received exemptions from clinical trials, as detailed in Table 2.

**TABLE 2:** Requirements of the evidence supporting the TCM approval from 2013 to 2024.

	Years			Registration categories before 2020			Registration categories after 2020					
	Pre-2020 (*n* = 33)	Post-2020 (*n* = 36)	*P*-value	Class 5 (*n* = 4)	Class 6^a^ (*n* = 29)	*P*-value^c^	Class 1.1^a^ (*n* = 18)	Class 1.2 (*n* = 4)	Class 2.2 (*n* = 1)	Class 3.1 (*n* = 8)	Class 3.2 (*n* = 5)	*P*-value^c^
**TCM theory**			**< 0.001**			**NA**						**0.008**
Yes	0 (0)	32 (88.9)		0 (0)	0 (0)		17 (94.4)	1 (25)	1 (100)	8 (100)	5 (100)	
No	33 (100)	4 (11.1)		4 (100)	29 (100)		1 (5.6)	3 (75)	0 (0)	0 (0)	0 (0)	
**Human use experience^b^**												
Ancient classic formulas	6 (18.2)	13 (36.1)	0.432	0 (0)	6 (20.7)	0.002	0 (0)	0 (0)	0 (0)	8 (100)	5 (100)	< 0.001
Clinical experience formulas	18 (54.5)	16 (44.4)		0 (0)	18 (62.1)		15 (83.3)	0 (0)	1 (100)	0 (0)	0 (0)	
Medical institution TCM preparations	2 (6.1)	1 (2.8)		0 (0)	2 (6.9)		1 (5.6)	0 (0)	0 (0)	0 (0)	0 (0)	
Pharmacology-based screening of TCM	7 (21.2)	6 (16.7)		4 (100)	3 (10.3)		2 (11.1)	4 (100)	0 (0)	0 (0)	0 (0)	
**Clinical trial phase**			**< 0.001**			**< 0.001**						**< 0.001**
Phase I, II, III	4 (12.1)	5 (13.9)		4 (100)	0 (0)		1 (5.6)	4 (100)	0 (0)	0 (0)	0 (0)	
Phase II, III	29 (87.9)	16 (44.4)		0 (0)	29 (100)		16 (88.8)	0 (0)	0 (0)	0 (0)	0 (0)	
Phase III	0 (0)	2 (5.6)		0 (0)	0 (0)		1 (5.6)	0 (0)	1 (100)	0 (0)	0 (0)	
Real word study	0 (0)	5 (13.9)		0 (0)	0 (0)		0 (0)	0 (0)	0 (0)	0 (0)	5 (100)	
Clinical waiver	0 (0)	8 (22.2)		0 (0)	0 (0)		0 (0)	0 (0)	0 (0)	8 (100)	0 (0)	

^a^The registration classification of Chinese medicines is based on the registration classification of the year of declaration. To more clearly compare the differences between Chinese medicines of different registration classes pre-2020 and post-2020, we classify the Chinese medicines of Class 6.1 approved for marketing in 2022, namely, Qijiao Tiaojing Granules, into Class 1.1 and conduct a statistical analysis in this way.

^b^Human use experience in human clinical practice, we categorize prescriptions derived from ancient formulas and ancient medical texts as classical formulas.

^c^*P*-values were calculated based on Fisher’s exact test. NA, not available.

### 3.3 Development pathways of new TCMs under the “three-in-one” evaluation and registration evidence system

**FIGURE 3:**
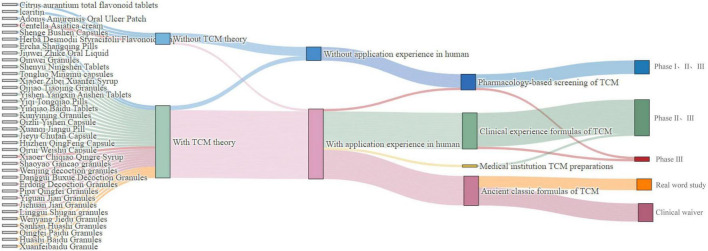
Clinical development pathways for post-2020 marketed drugs. TCM, traditional Chinese medicine.

Under the three-in-one evaluation and registration evidence system, discrepancies exist in the development pathways of new TCMs, as illustrated in the Figure 3. Firstly, for new TCMs without TCM theory and human use experience, such as Citrus aurantium total flavonoid tablets, claritin, and Adonis Amurensis Oral Ulcer Patch, which were developed through modern pharmacological research methods, it was necessary to first screen for active substances, identify and verify the target sites of action, clarify the material basis, and conduct Phase I, II, and III clinical trials. Secondly, For new TCMs that were based on TCM theory but lacked human use experience, such as Herba Desmodii Styracifolii Flavonoids Capsule, which was a preparation made from Guang Qian Total Flavonoid Extract, extracted from Guang Qian Herb, it was necessary to carry out exploratory clinical studies and confirmatory tests and provide complete clinical trial evidence for registration. Thirdly, in the case of TCMs, for which there was an absence of clear theoretical support, such as Centella Asiatica cream, the development of the drug was not based on the aforementioned theoretical system. Instead, it relied primarily on the accumulation of practical experience. The drug has accumulated some human use experience through international multi-center clinical trials. In order to further validate its safety and efficacy, the marketing approval process was planned to be completed through Phase III clinical trials. Finally, TCM compound preparations, developed on human use experience, were mainly formulated from ancient classical formulas or clinical experience formulas. With sufficient TCM theoretical support, the maturity and reliability of the human use experience can be used to decide whether to carry out clinical trials and the specific stages of the trials. If the human use experience can provide sufficient supporting evidence regarding drug safety, such as Ercha Shangqing Pills and Jiuwei Zhike Oral Liquid, the TCMs can enter directly into phase II and phase III clinical trials. For ancient classic prescriptions with long-history clinical practice, such as Yiguan Jian Granules, Jichuan Decoction Granules, etc., the requirement for clinical trials can be exempted if specific conditions were met. Furthermore, ancient classic formulas with rich clinical practice, such as QingFei Paidu Granules, HuaShi Baidu Granules, etc., could be marketed directly through real-world evidence.

### 3.4 Pivotal clinical trials and real-world studies supporting the TCM approval for marketing

#### 3.4.1 Pivotal clinical trials

The development of new TCMs should be grounded in “meeting clinical needs and discovering clinical value” (25). Five situations were listed in the Specialized Management Regulations for the Registration of TCMs to evaluate the clinical value of the TCMs, including recovery from disease, delayed disease progression, the improvement of the condition or symptoms, the improvement of the patient’s disease-related body functions or quality of life and others (increasing efficacy and reducing toxicity of TCMs through use in combination with chemical pharmaceuticals, or reducing the dosage of chemical pharmaceuticals with obvious side effects).Our data indicated that new TCMs were clinically focused on reducing or eliminating clinical symptoms (17, 30.3%) and improving the patient’s body functions related to the disease (16.28.6%). In post-2020 period, new TCM development is gradually oriented toward higher clinical value, with significant increases in delayed disease progression (17.4% vs. 15.2%) and disease recovery (30.4% vs. 12.1%) compared to pre-2020.

As shown in Table 3 and Supplementary Table 6, the pivotal clinical trials underpinning the approval of the 56 TCMs between 2013 and 2024 were Phase III clinical studies. The majority of these trials were designed as randomized, double-blind, multi-center clinical trials and median value of enrolment was 478. However, a drug (Centella Asiatica cream) imported from Taiwan, China was the only new TCM to have undergone international multi-center clinical trials. Notably, the proportion of trials incorporating placebo control settings significantly increased from 15.2% pre-2020 to 65.2% post-2020 (*P* < 0.001). In terms of efficacy evaluation, a substantial number of the new TCMs utilized surrogate indicators (17, 30.4%) and patient-reported outcomes (PROs) (19, 33.9%) to assess their efficacy and safety. However, using TCM syndromes and endpoints as efficacy evaluation indicators was less prevalent. Especially in the post-2020, none of the TCMs used TCM syndromes and endpoints as main efficacy indicator.

**TABLE 3:** Clinical value and Pivotal clinical trial design for new TCM, 2013–2024.

	No. (%)			
	Total (*n* = 56)	Pre-2020 (*n*-33)	Post-2020 (*n* = 23)	*P*-value
**Clinical value^a^**				**0.093**
Recovery from disease	11 (19.6)	4 (12.1)	7 (30.4)	
Delayed disease progression	8 (16.1)	5 (15.2)	4 (17.4)	
Improvement of the condition or symptoms	16 (28.6)	11 (33.3)	5 (21.8)	
Improvement of the patient’s disease-related body functions or quality of life	17 (30.3)	10 (30.3)	7 (30.4)	
Others	3 (5.4)	3 (9.1)	0 (0)	
**Phase III**				**NA**
Yes	56 (100)	33 (100)	23 (100)	
No	0 (0)	0 (0)	0 (0)	
**Enrollment, median (IQR)**				0.77*
No. of patients	478 (447–510)	478 (456.548)	478 (432–480)	
**Multicenter**				**0.411**
Domestic multi-center	56 (100)	33 (100)	22 (95.7)	
MRCT	0 (0)	0 (0)	1 (4.3)	
**Randomization**				**NA**
Yes	56 (100)	33 (100)	23 (100)	
No	0 (0)	0 (0)	0 (0)	
**Blinding**				**0.411**
Single-blind/open label	1 (1.8)	0 (0)	1 (4.3)	
Double-blind	55 (98.2)	33 (100)	22 (95.7)	
**Comparator**				**< 0.001**
Placebo	20 (35.7)	5 (15.2)	15 (65.2)	
Active	25 (44.7)	20 (60.6)	5 (21.7)	
Placebo and active	11 (19.6)	8 (24.2)	3 (13.1)	
**Main efficacy indicators^b^**				**0.143**
TCM syndromes	2 (3.6)	2 (6.1)	0 (0)	
Endpoint indicators	18 (32.1)	8 (24.2)	10 (43.5)	
Surrogate indicators	17 (30.4)	13 (39.4)	4 (17.4)	
PRO	19 (33.9)	10 (30.3)	9 (39.1)	

*P-value was calculated based on Mann–Whitney U tests. *P*-values were calculated based on Fisher’s exact test. NA, not available.

^a^Others include increasing the efficacy and reducing the toxicity of TCMs through use in combination with chemical pharmaceuticals, reducing the dosage of chemical pharmaceuticals with obvious side effects and other situations.

^b^In the clinical efficacy evaluation indexes, TCM syndromes were categorized as TCM syndromes evaluation indexes. Important outcome ratios, such as death, cure, and recurrence, were used as endpoint indexes. Biological indexes, symptoms, and functional evaluations were used as surrogate indexes, and scales were categorized as patient-reported outcomes. PRO, patient-reported outcomes.

#### 3.4.2 Real-world studies

Real-world studies (RWS) are studies in which multiple data are obtained in real clinical, community, or home settings. The objective of RWS is to evaluate the true impact of a treatment measure on patient health (26). According to our statistics, five TCMs (treatment for COVID-19) in post-2020 were approved on the basis of evidence from real-world studies. A retrospective observational study of Qingfei Paidu granules, with 3,715 cases from more than 60 medical institutions in 28 provinces, from human use empirical evidence to support NDA approval (27). The drug’s clinical efficacy and safety in patients with HuaShi Baidu Granules were systematically evaluated by integrating retrospective studies, prospective non-randomized controlled studies, and pragmatic clinical studies (PCTs). The evidence from these studies covered a range of multidimensional clinical scenarios, supporting the drug’s approval. XuanFei Baidu Granule was a real-world data mining and analysis that is finally transformed into evidence of human use experience that meets the requirements for the declaration of a new TCM. Sanhan Huashi Granules have been comprehensively validated through real-world data and randomized controlled clinical trials, providing strong evidence for its approval. Wenyang Jiedu Granules were approved for marketing in 2024 after thousands of clinically validated results showed favorable efficacy in reducing disease symptoms (see Supplementary Table 7).

### 3.5 Development duration and review time of the new TCMs

**FIGURE 4:**
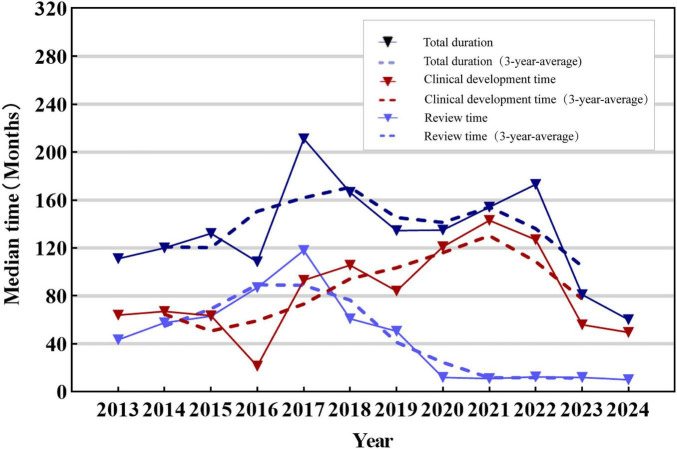
Median time to approval of new TCMs, 2013–2024. The solid line in the figure represents the trend of development, review and total duration of new TCMs, while the dotted line represents the trend of development, review and total duration of new TCMs from the three-year average. The colors blue, red and dark blue are used to represent the review time, development time and total duration, respectively.

23 TCMs classified were excluded from the analysis due to the unavailability of the exact date of acceptance. 54 new TCMs approved between 2013 and 2024 can be accessed with the exact dates of clinical trial approval and marketing application submission, enabling the calculation of development times. As shown in Figure 4, the total median development time of marketed TCM was found to be 121 months (IQR: 100–151.75 months), and clinical development time fluctuated over the year. When evaluated over a three-year average, the overall trend was an initial increase followed by a subsequent decrease. The analysis results demonstrated that the median review time for new TCMs on the market from 2013 to 2024 was 40 months (IQR: 12.75–61.5 months). Over time, the review time for TCMs demonstrated fluctuations, although the overall trend was one of decrease. However, the overall trend in total duration was relatively flat.

**FIGURE 5:**
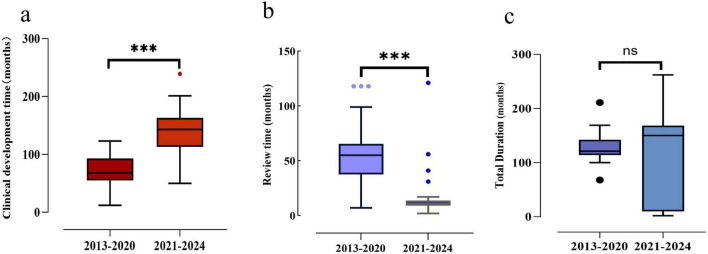
Development time, review time and total duration of approved new TCMs, 2013–2024. **(a)** Development time comparison between the two time periods. **(b)** Review time comparison between the two time periods. **(c)** Total duration time comparison between the two time periods. Box plots indicate interquartile ranges in shade areas and maximum and minimum values in whiskers, and the dots indicate outliers. ***p < 0.001; ns, no significant values.

As illustrated in Figure 5, the median clinical development time for new TCMs post-2020 (median: 143 months, IQR: 113–163 months) was found to be significantly longer than that observed pre-2020 (median: 68 months, IQR: 55–93 months). In contrast, the median review time for new TCMs post-2020 (medians: 11 months, IQR: 9–14 months) was markedly shorter than that observed pre-2020 (medians: 55 months, IQR: 37.5–65.5 months). There was a statistically significant difference between the two groups (*P* < 0.001). However, the total duration of TCMs in both phases did not exhibit a notable discrepancy.

The development time and review time in post-2020 were analyzed. The data demonstrated that there were substantial variations in the development and review time of new TCMs of differing registration categories. Specifically, the development time for new TCM in class 1.2 (e.g., Citrus aurantium total flavonoid tablets, Herba Desmodii Styracifolii Flavonoids Capsule, etc.) was generally longer than that for new TCM in classes 1.1 (e.g., Yiqi Tongqiao Pills, Yinqiao Qingre Tablets), 2 and 3. A general observation of the review process indicated that the time allotted for evaluating the class 3 ancient classical formulas was less than that allocated for the other classes. The median development time for the new TCMs was approximately 143 months, while the median review time was approximately 10 months. As shown in Supplementary Figures 3, 4.

## 4 Discussion

This study observed that the number of new TCMs remained relatively high until 2015. However, by 2017, this number had precipitously declined to single-digit approvals. Prior to 2015, the Chinese government promoted the notion that new TCMs should adhere to the same clinical trial standards as chemical pharmaceuticals, necessitating robust clinical evidence, such as RCTs, and should be assessed according to standardized guidelines and efficacy criteria. Consequently, the development of new TCMs over an extended period has been characterized by a low success rate, substantial investment, prolonged development cycles, and elevated risk. These factors have contributed to the prevalence of non-standardized practices among numerous TCM enterprises aimed at generating data that complies with requisite standards. Following 2015, the NMPA implemented the “Announcement on Conducting Self-Inspection and Verification of Drug Clinical Trial Data” to address non-compliance in clinical trial data (28). This initiative resulted in the withdrawal of numerous NDAs for TCMs by various companies, consequently leading to a marked decline in the number of new TCMs (10). However, the data presented in this study indicated a gradual increase in the approval of new TCMs for marketing subsequent to the reform of the TCM review and approval in 2020. This trend suggests that the reform has been effective in facilitating the approval process for TCMs.

Between 2013 and 2024, the majority of new TCMs in China were compound preparations, constituting 88.3% compared to 11.7% for single-prescription. Compound preparations are more effective in treating a range of diseases due to the synergistic interactions among multiple Chinese medicinal ingredients, thereby enhancing therapeutic efficacy for specific conditions (29). Historically, compound preparations have been the predominant focus in the development of new TCMs. Moving forward, research and development in this field will continue to prioritize compound preparations, with an emphasis on innovation grounded in inheritance.

In recent years, the Chinese government has approved most new drugs as chemical and biological products, with indications spanning anti-tumor, anti-infection, dermatological and rare disease treatments (30–32). Nevertheless, the number of approvals for TCMs in the aforementioned therapeutic areas was relatively limited and primarily concentrated in regions lacking efficacious therapeutic chemicals and biological products. In contrast to chemical drugs and biological products, TCM therapy emphasizes the comprehensive regulation of human physiological processes, thereby conferring certain advantages in treating chronic diseases. For example, TCM theory posits that the etiology of respiratory diseases is linked to the malfunction of multiple internal organs, including the lungs, spleen, and kidneys. Consequently, with its multi-component, multi-pathway, and multi-target mechanism of action, TCM is frequently capable of addressing both the presenting symptoms and the underlying causes (33). Furthermore, TCM is widely used in the neurological field. This is because numerous TCM ingredients possess sedative and antidepressant functions, which have been demonstrated to have beneficial therapeutic effects on insomnia, anxiety, depression, and other neurological disorders (34).

A review of relevant studies indicated that between 2016 and 2018, only 1% of TCMs were included in the PR designations (35), and only 3% of TCMs were marketed through expedited programs from 2019 to 2021 (36). Consequently, the expedited programs were less frequently employed for new TCMs than for chemical and biological products. China’s expedited programs focus on supporting drugs that are urgently needed in clinical or in public health, for which there are no effective treatments or insufficient effective treatments, especially anti-tumor drugs and rare disease drugs (37, 38).The findings of this study demonstrated that the majority of new TCMs in China were concentrated in the field of chronic diseases. This made it challenging for them to meet the applicable conditions of the expedited programs, resulting in a lower rate of expedited approvals. The new TCMs marketed under the expedited programs usually had obvious clinical advantages. For example, the NMPA included the Shaoma Zhijing granule on the PR list in September 2018 due to the medication’s evident therapeutic advantages and suitability for children. Epimedium Soft Capsule, an original TCM independently developed in China, has been granted CA and PR. It was a promising new treatment option for liver cancer patients and was approved for marketing in 2022. Additionally, three products that were superior varieties for the treatment of COVID-19 were approved for marketing on an emergency basis through SRAP in 2021. It is suggested to develop specialized expedited programs for TCMs, with the aim of enabling a greater number of TCMs to benefit from the policy advantages associated with expedited programs.

Compared with other types of drugs, the most important feature of TCMs is that it has undergone a long period of clinical practice, contain a wealth of application experience in clinical use, and follow the development path of “clinical-laboratory-clinical” (39). The existing evaluation and registration evidence system for chemical drugs is ill-equipped to adequately reflect the research and development rules of traditional Chinese medicines guided by TCM theories and with human use experience. Consequently, making a scientific assessment of their clinical value is impossible. Since 2020, the Chinese government has been focusing on the role of TCM theory and human use experience in developing TCMs. This has resulted in the construction of a “three-in-one” evaluation and registration evidence system designed to facilitate the development of new TCMs. Under this evidence system, the approval of new TCMs relies not only on a single clinical trial but also on TCM theory and empirical data from human use experience. TCMs with differing registration classifications exhibit variations in their theoretical foundations and experiential applications in human subjects. As a result, the evidentiary requirements from clinical trials are subject to fluctuation. In summary, the endorsement of TCM theory and substantial experiential evidence from human use may lessen the necessity for stringent clinical trial evidence. Conversely, more extensive clinical trial evidence is required to substantiate the approval of TCMs. The ongoing advancement and refinement of the “three-in-one” evaluation and registration evidence system necessitate the backing of a comprehensive theoretical framework. This includes the analysis of clinical demand for the development of new TCMs, the design of clinical positioning for research and development in TCMs, the evaluation of the value of new TCMs, the formulation of clinical research and development strategies grounded in human use experience, the exploration of efficacy outcome indicators according with the characteristic of TCMs, and the establishment of an evidence-based system rooted in human experience.

Under the “three-in-one” evaluation and registration evidence system, there are multiple development pathways for new TCMs (40). Collecting and summarizing human experience information to form data and evidence that can be used for evaluation is an important part of implementing the “three-in-one” evaluation and registration evidence system. On the one hand, a comprehensive review of empirical evidence regarding the application experience in humans of TCM facilitates a timely evaluation of the clinical positioning of new TCM interventions. This assessment encompasses the magnitude of clinical benefits, the appropriateness of dosage and administration duration, and the severity and incidence of both anticipated and unforeseen adverse reactions. Consequently, such an evaluation mitigates the risk associated with late-stage research and development. On the other hand, these analyses can provide support for the design of clinical trial protocols for new TCMs, encompassing aspects such as sample size estimation, selection of control drugs, inclusion and exclusion criteria, criteria for evaluating clinical efficacy, safety evaluation indices, and the scheduling of visits and follow-ups (41). Based on the robustness of evidence regarding human use experience and compliance with relevant requirements for the registration and review of new TCMs, approvals may be granted to conduct clinical trials, to be exempt from pharmacodynamic studies, or to be exempt from clinical trials. For single-prescription, these drugs are relatively well-characterized but lack human use experience. Consequently, exploratory trials and confirmatory trials are generally necessary to ascertain the safety and efficacy of the new TCMs. For compound preparations, it is still necessary to conduct exploratory trials and confirmatory randomized controlled clinical trials in the absence of sufficient application experience in humans. Conversely, exploratory trials may be omitted when there is substantial application experience in humans. In the case of ancient classic formulas, these new drugs typically possess extensive and longstanding histories of application experience in humans, thereby obviating the need for clinical trials. High-quality empirical evidence of application experience in humans can be directly utilized as pivotal support for marketing, contingent upon dialog with regulatory authorities (42). The systematic application of evidence from human use experience is crucial in the advancement of efficacious new TCMs. This necessitates that practitioners rigorously adhere to and standardize clinical diagnostic and therapeutic procedures, meticulously gather and systematize empirical data from clinical practice, and proactively employ contemporary scientific methodologies, including artificial intelligence and data science, to undertake high-caliber research on human experiences.

Clinical value is the core starting point of all new TCM research and development, so the research and development of new TCM drugs should follow the R&D idea oriented by TCM’s clinical value (43, 44). Clinical value for chemical or biological drugs means drugs are included in any following circumstances: The first, in the absence of effective prevention and treatment methods, the drug demonstrates substantial clinical efficacy in critical clinical outcomes relative to placebo or historical controls, supported by robust evidence. The second, compared with existing treatment methods, this drug has more significant or important therapeutic effects. The third, compared with existing treatment methods or historical controls with good evidence, the combination of this drug with existing treatment methods produces more significant or important therapeutic effects. The fourth, the existing treatment methods can only treat the symptoms of the disease, while this drug can treat the cause and has significant clinical efficacy. It can reverse or inhibit the progression of the disease and may bring sustained clinical benefits, avoiding consequences that seriously endanger life or significantly affect quality of life. The last, compared with currently irreplaceable treatment methods, the efficacy of the drug is comparable, but it has significant safety advantages. The drug is expected to replace existing treatment methods or provide important supplements to existing treatment methods (45, 46). In contrast to chemical or biological drugs, the clinical value of TCMs is predominantly centered on their diagnostic and therapeutic applications, as well as the anticipated outcomes, which are grounded in the cultural understanding of life and health. This value is reflected in the holistic benefits to patients, including life preservation, health restoration, and pain alleviation. It is recommended that a more comprehensive set of multidimensional clinical value evaluation indicators be developed for TCMs. These should encompass assessments based on the characteristics of the user population, comparisons with existing treatment modalities, and evaluations aligned with specific therapeutic objectives.

Our date indicated that the clinical development of TCMs was predominantly conducted in mainland China. This observation aligns with the previously noted fact that the majority of TCMs were produced domestically. This suggests that the degree of internationalization of TCMs remains comparatively limited. Looking forward, it is imperative to further investigate pathways for the global development of TCMs, particularly by enhancing the implementation of international multicenter clinical trials. A notable increase in the percentage of placebo use was observed in clinical trials of new TCMs post-2020 compared to the period pre-2020. Except for compound preparations of TCM that meet the requirements for exemption from clinical trials and can be approved based on human use experience, the development of new TCMs, particularly single-prescription of TCMs, must be subject to clinical trials to verify their efficacy and safety. Furthermore, priority is given to the use of randomized controlled multi-center clinical study design (47). In contrast to the clinical assessment of chemical drugs and biological products, the clinical investigation of new TCMs is predominantly founded upon reflecting Chinese medicine’s dialectical thinking and distinctive therapeutic modalities. Therefore, changes in TCM’s symptoms and quality of life are often involved in the evaluation criteria. Many of these alterations are non-objective indicators, which are more vulnerable to subjective influences by clinicians or subjects during efficacy assessment than objective indicators (48, 49). Implementing placebo controls can mitigate the impact of these subjective interferences to a certain extent. In 2023, the NMPA issued Special Regulations on Registration and Management of Traditional Chinese Medicines, which proposed that clinical trials of new TCMs should encourage the preferred use of a placebo or placebo control loaded with the underlying treatment. The findings of this study also demonstrated that following 2020, most clinical trials of new TCMs were placebo-controlled, thereby corroborating the therapeutic efficacy of the drugs themselves (7).

The evaluation of the clinical efficacy of new TCMs was predominantly based on patient-reported outcomes (PROs) (33.9%), with endpoint indicators and surrogate indicators accounting for 32.1% and 30.4%, respectively. Additionally, two studies (3.6%) employed TCM syndrome evaluation. Of these, the clinical endpoint and surrogate endpoint were objective evaluation indicators. Subsequent to 2020, the proportion of clinical endpoints employed increased, while the proportion of surrogate endpoints decreased. TCM syndrome is defined as the reflection of the specific internal and external environments at a given stage in the disease occurrence and evolution process, as well as the individual patient at that time. It is a non-objective evaluation criterion. Due to the absence of uniformity in evaluation standards, the NMPA has not advocated the utilization of TCM syndromes as clinical efficacy evaluation indicators in recent years (50). The data indicated that post-2020, no new TCMs had been granted marketing approval via a single standard for evaluating TCM syndromes. However, there has been a notable increase in the utilization of PROs. The data above illustrated that scientific, standardized, and patient-centered review concepts are increasingly pivotal in evaluating novel TCMs. It is important to acknowledge that the majority of scales presently employed in critical efficacy evaluations are adapted from those utilized for assessing chemical drugs and biologics. Consequently, there is a paucity of scales specifically designed for evaluating the efficacy of TCMs (51, 52). Hence, future efforts should focus on developing measurement tools that are grounded in the clinical and scientific contexts of TCMs, integrating modern scientific and technological advancements to establish a robust framework for assessing the therapeutic efficacy of Chinese medicine (53).

Between 2013 and 2024, five TCMs were marketed based on evidence of real-world application experience in humans. The development process of these drugs fully utilized real-world data combined with long-accumulated clinical experience, verifying their efficacy and providing a new pathway of the innovative development of Chinese medicine. Compared with the conventional RCT, the notion of RWS aligns with TCM’s systematic and multidimensional dialectical thinking and the tradition of empirical medicine of “from the clinic, to the clinic” (54, 55).Regarding holistic concepts, RWS can comprehensively depict the mechanism of action and overall effects of TCM in the complex human environment through large-scale data collection and analysis. The scope of TCM has evolved beyond the confines of a single target or symptom, encompassing a comprehensive regulatory effect on multiple systems and levels of the human body from a macroscopic perspective. For instance, by monitoring alterations in patients’ health status over time, the long-term benefits of Chinese medicines in enhancing overall health and quality of life and preventing disease recurrence can be evaluated. Regarding evidence-based treatment, RWS can fully explore the impact of individual differences on the effectiveness of Chinese medicine treatment (56). Utilizing advanced data analysis technology, a comprehensive evaluation of the patient’s demographics, including age, gender, geographical location, physical condition, and underlying diseases, is conducted. This multifaceted analysis aims to precisely ascertain the response patterns of diverse individuals to Chinese medicine treatment (57).In the future, greater emphasis should be placed on implementing real-world research in the clinical practice of traditional Chinese medicine. Furthermore, for drugs already used in human clinical trials, efforts should be made to integrate real and randomized clinical trials to explore new avenues for clinical research and development (58).

Following the reform of the TCM development and approval system, the exemption of pharmacodynamic studies and clinical trials under the “three-in-one” evaluation and registration evidence system has significantly reduced the development duration for new TCMs. Subsequently, following the year 2020, to encourage the development of new, clinically efficacious TCMs, China has implemented an approval system that aligns with the distinctive attributes of TCMs. For example, applications for new TCMs for the prevention and treatment of serious and rare diseases will be accorded PR status, and medicines for treating severe, life-threatening diseases for which no effective treatment is available may be approved with conditions. SRAP will be granted to TCMs marketed as essential for emergency response in a significant public health emergency (4). Furthermore, the State advocates the incorporation of real-world evidence to substantiate the approval of new TCMs while streamlining the approval process for traditional classical prescriptions. These review and approval systems and innovation incentives, in line with the characteristics of TCM, have shortened the review and approval time for TCMs to a certain. Conversely, the development time of TCM exhibits an inverse trend, it may indicate that the reduction in NDA review time is attributable to the enhancement of the registration management system and the standardization of drug clinical trials. It is recommended to develop a diversified development framework for new TCMs. This framework should emphasize the integration of innovative technologies, including network pharmacology, genetic modification, artificial intelligence, and synthetic biology, to advance the development processes of new TCMs. Concurrently, regulatory strategies should be enhanced by refining the technical guidance principles for the development of new TCM drugs and incorporating innovative benefit-risk assessment tools. These measures aim to improve the efficiency and expedite the development of new TCMs.

## 5 Conclusion

Through the implementation of various regulations, policies, and related measures, the reform of the development and approval for TCM has yielded significant outcomes. Notably, the establishment of the “three-in-one” evaluation and registration evidence system tailored to the unique characteristics of TCMs has positively influenced development and development of new TCMs. Despite these advancements, several challenges persist in the development and approval of new TCMs. First of all, the development and enhancement of the “three-in-one” evaluation and registration evidence system, alongside the execution of high-quality research on human use experiences and the standardization of evidence collection in this domain, present significant challenges that warrant careful consideration. Additionally, the internationalization of TCMs is still at a nascent stage, with activities predominantly confined to the domestic market and a notable deficiency in international multicenter clinical trials. Furthermore, while the clinical value assessment of TCMs is more “patient-centered” compared to biological or chemical drugs, there is a paucity of objective indicators in its application. The implementation of the expedited programs for the development and approval of TCMs is constrained, and the reduction in development time for new TCMs following the reform is not substantial. The aforementioned issues not only present challenges to the advancement of reform in the development and approval system but also offer novel opportunities for the innovative development of TCMs.

## 6 Limitation

This study has the following limitations: Firstly, the limited sample size may affect the representativeness of the findings to the overall situation of new TCM development. Additionally, the lack of public access to information on IND applications and approvals of some TCM resulted in incomplete data on the length of the review, which to a certain extent affects the comprehensiveness of the evaluation of approval timeliness.

## Data Availability

The original contributions presented in this study are included in this article/Supplementary material, further inquiries can be directed to the corresponding author.
