# Supplementary material for: Promoting the development and approval of new traditional Chinese medicines in China: a pooled analysis of data from 2013 to 2024
Source: Front Med (Lausanne). 2025 Jun 16;12:1559703. doi: 10.3389/fmed.2025.1559703 (PMC12206838; doi:10.3389/fmed.2025.1559703)
Supplement: Supplementary file 1 [file Data_Sheet_1.docx]

Supplementary Material

# Supplementary Figures


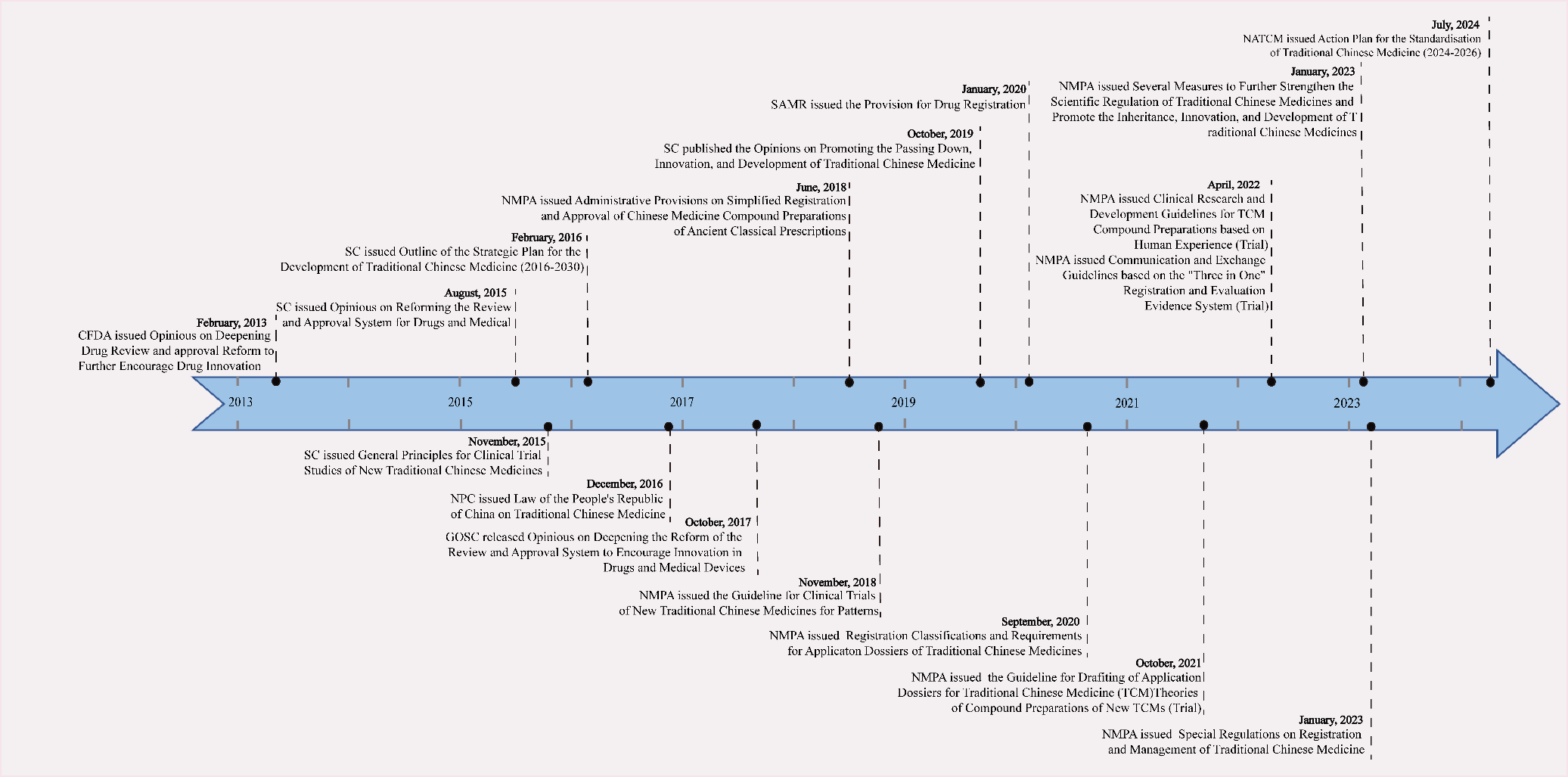


## Supplementary Figure 1. Timeline of major TCM regulatory reform initiatives in China.

Abbreviations:
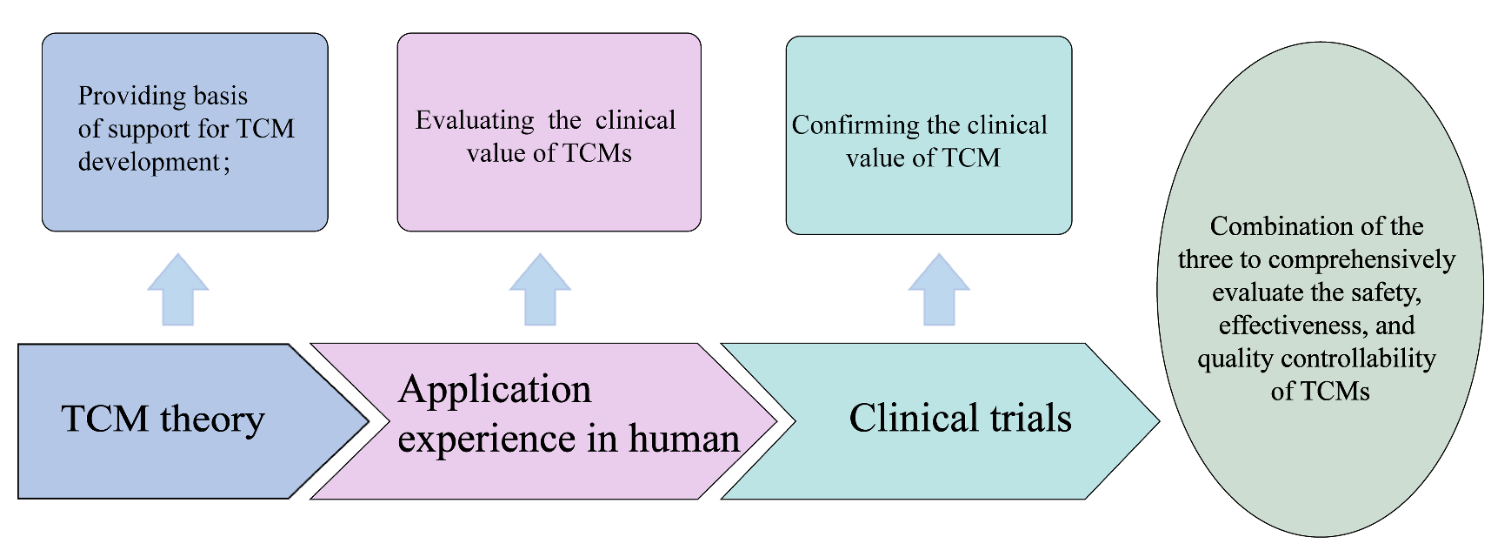
CFDA, China Food and Drug Administration (changed to the National Medical Products Administration in 2018); SC, State Council; GOSC, General Office of the State Council; NMPA, National Medical Products Administration; NPC, National People's Congress; SAMR, State Administration of Market Regulation. NATCM, National Administration of Traditional Chinese Medicine.

## Supplementary Figure 2. Evidence system framework for the ‘three-in-one’ review of TCMs.

Abbreviations: TCM, traditional Chinese medicine.


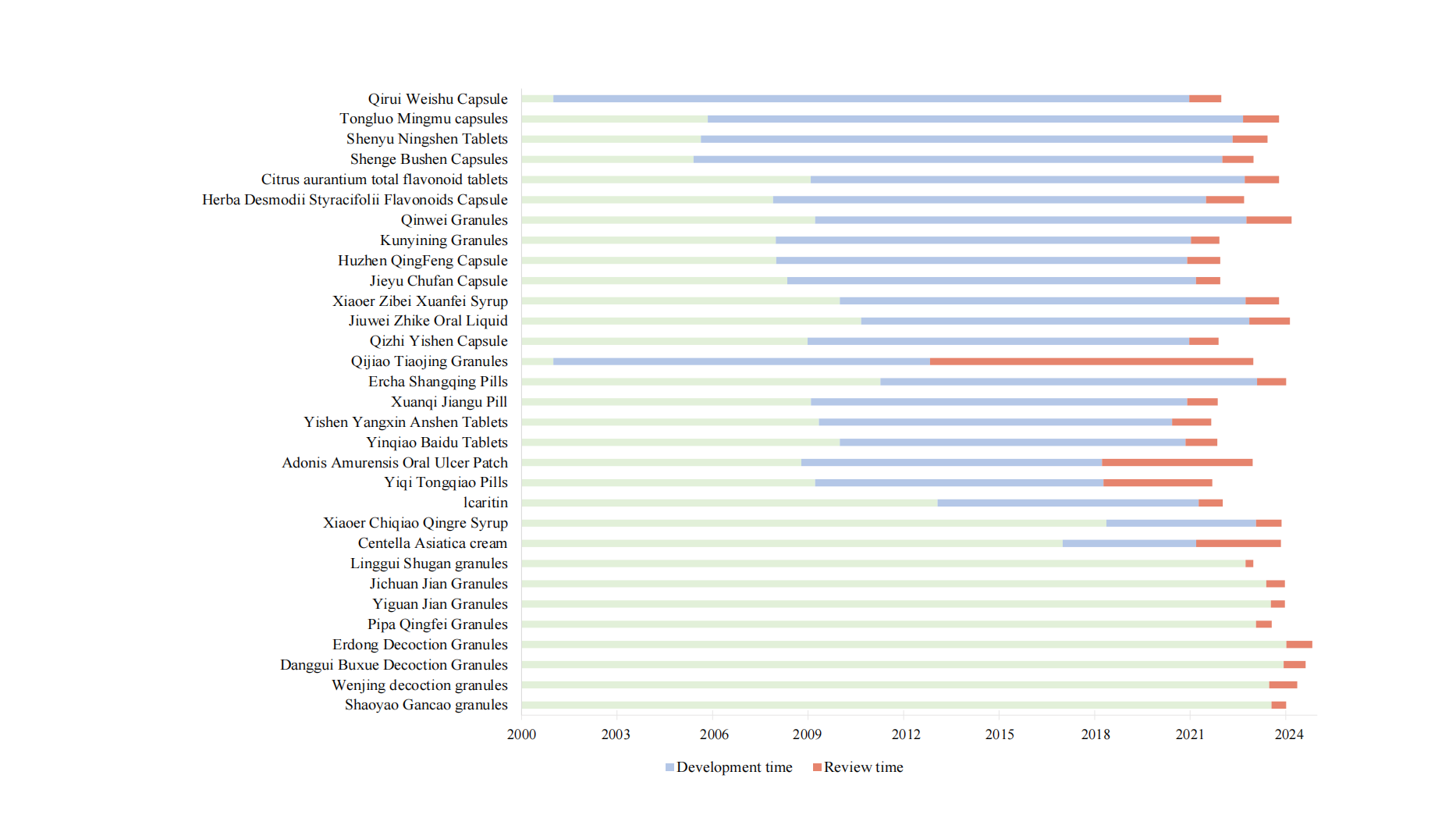


## Supplementary Figure 3. Comparison of development and review timelines for new traditional Chinese medicines approved in 2020.


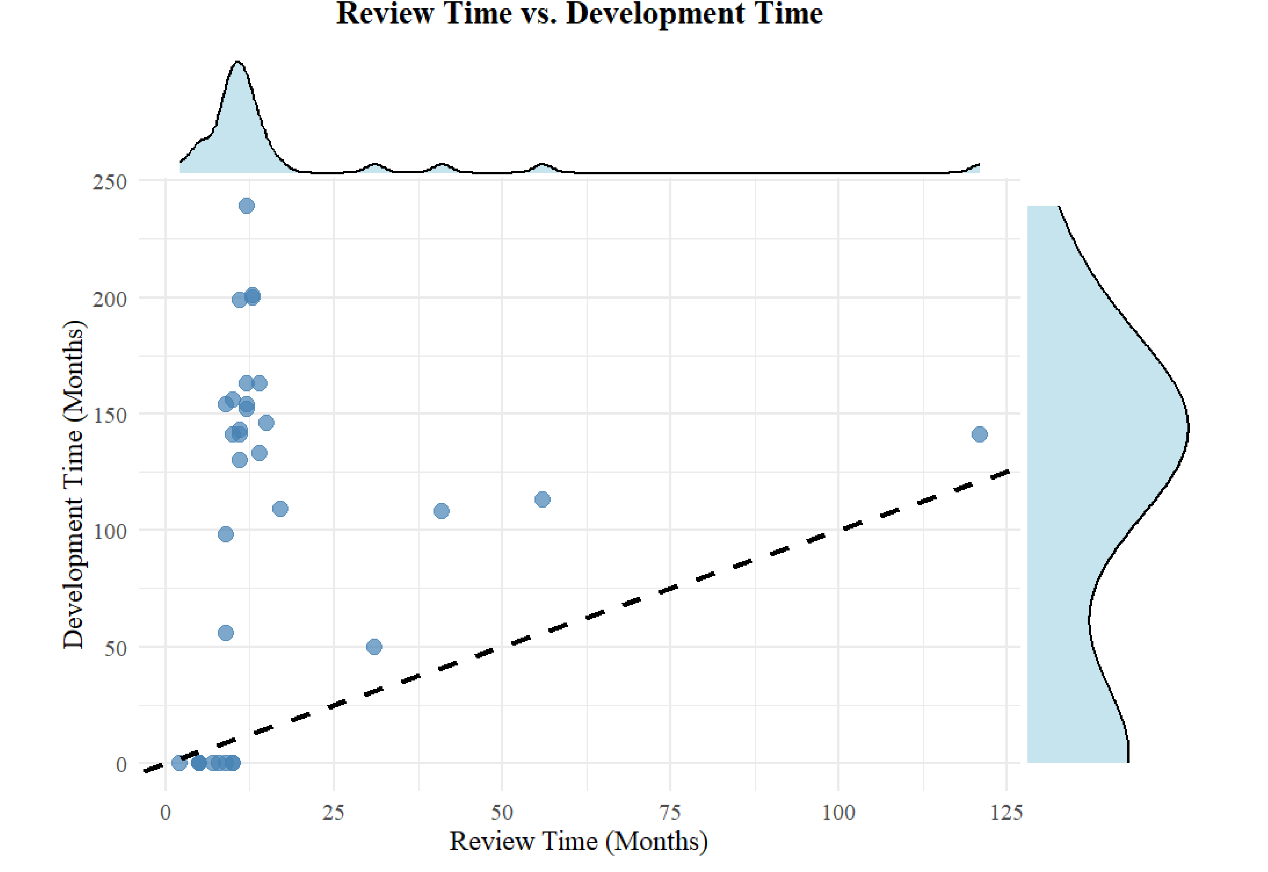


## Supplementary Figure 4. Central tendency of development and review times for 2020 marketed new TCMs.

Note: The black dotted line in the figure represents the curve y = x.

# Supplementary Tables

## Supplementary Table 1. Categories of new traditional Chinese medicine registration in various editions of Provisions for Drug Registration in China.

| Registration classification | Provisions for Drug Registration, June 2007 | Provisions for Drug Registration, March 2020 |
| --- | --- | --- |
| 1 | Active ingredients and their preparations extracted from plants, animals and minerals., or other substance which have not been marketed in China | Innovative TCMs  1.1 Compound preparations of TCMs, which refer to preparations made with multiple prepared slices/decoction pieces and extracts, ete. based on TCM theories.  1.2 Extracts obtained from single plant, animal or mineral materials and their preparations.  1.3 New TCM crude drugs and their preparations, which refer to TC crude drugs not included in the national drug standards, national drug registration standards or provincial standards, and their preparations, as well as new medicinal parts of TCM crude drugs included by the above standards, and their preparations. |
| 2 | Newly discovered crude drugs and their preparations | Modified new TCMs  2.1 Preparations with a changed administration route and/or drug-absorbing sites to the marketed TCMs.  2.2 Preparations with a changed dosage form to the marketed TCMs, without changing the administration route.  2.3 TCMs with added functions and indications.  2.4 TCMs with changes in manufacturing process or excipients to marketed TCMs, causing substantial changes in medicinal substances or their absorption and utilization. |
| 3 | New substances of existing Chinese crude drugs | Complex-formulated TCM preparations derived from ancient classical formulas and from formulas prescribed by prestigious veteran practitioners.  3.1 Compound preparations of TCMs managed in accordance with the Directory of Ancient Classic Formulas of Traditional Chinese Medicines.  3.2 Other compound preparations of TCMs originated from ancient classic formulas, including compound preparations of TCMs originated from ancient classic formulas not listed in the Directory of Ancient Classic Formulas of Traditional Chinese Medicines and those with addition or subtraction of ingredients, based on ancient classic formulas. |
| 4 | New medicinal parts of existing crude drugs or their preparations | TCMs with identical name and formula (to marketed TCMs) |
| 5 | Active fractions and their preparations extracted from plants, animals, minerals, or other substances that have not been marketed in China |  |
| 6 | Complex-formulated TCM other substances that have not been marketed preparations derived from ancient |  |
| 7 | Preparations with change in route of administration of the TCM or natural drugs already marketed in China |  |
| 8 | Preparations with change in dosage form of the TCM or natural drugs already marketed in China |  |
| 9 | Generic drugs |  |

Abbreviations: TCM, traditional Chinese Medicine.

## Supplementary Table 2. Explanation of terms in the text.

| Terms | Definition |
| --- | --- |
| traditional Chinese medicine (TCM) | TCMs refer to medicinal substances and their preparations used under the guidance of the theory of TCM in China. |
| Innovative TCMs | Innovative TCMs, which refer to the new TC formula preparations that have clinical value and have not been marketed overseas, and whose formulas are not included in the national drug standards, national drug registration standards or Directory of Ancient Classic Formulas of Traditional Chinese Medicines issued by the National Administration of Traditional Chinese Medicine. |
| Improved new TCMs | Improved new TCMs, which refer to the preparations that change the administration routes and/or dosage forms to marketed TCMs, with clinical application advantages and characteristics, or add functions and indications, etc. |
| Compound preparations of TCMs originated from ancient classic formulas | Compound preparations of TCMs originated from ancient classic formulas, which refer to the prescriptions recorded in ancient TCM classics that conform to the regulations of the Law of the People's Republic of China on Traditional Chinese Medicines and are still used currently in practice, with confirmed efficacy and significant characteristics and advantages. |
| Clinical experience formulas | Clinical experience formulas of traditional Chinese medicine (TCM) is an effective prescription for treating certain diseases or symptoms summarized by TCM doctors in practice. |
| Medical institution TCM preparations | Medical institution TCM preparations are fixed-prescription preparations specially formulated by medical institutions to meet clinical needs. |
| Pharmacology-based screening of TCM | Prescriptions based primarily on animal researches and other research findings. |
| TCM theory | The theory of TCM is a theoretical system with distinctive traditional cultural characteristics under the guidance of ancient philosophical thought, guided by the holistic view and framed by yin and yang and the five elements, covering many aspects of health concepts, disease awareness, medication concepts, diagnosis and treatment and value orientation of TCM, and forming the characteristics of diagnosis and treatment, and flexible and balanced prescription and use of medication. |
| Application experience in humans | The application experience in humans is typically derived from clinical practice and is characterized by regularity, reproducibility, and clinical significance. |
| Three-in-one | The text describes an evidence-based framework for evaluating TCMs that synthesizes TCM theory, application experience in humans, and clinical trial data. |
| TCM single preparation | Single-prescription of TCMs are extracts obtained from a single plant, animal, or mineral materials and their preparations. |
| TCM compound preparations | Compound preparations of TCMs refer to preparations made with multiple prepared slices/decoction pieces and extracts based on TCM theories. |
| Real-world research | Real-world research refers to the process of collecting real-world data related to the health of research subjects in a real-world setting in response to a predefined clinical question, and analyzing them to obtain information on drug use and potential benefits/risks, with data from health information systems, health insurance systems, disease registries, and adverse drug reaction monitoring systems, among others. |
| TCM Syndrome | TCM syndrome is a reflection of the essence of a disease at a certain stage in its occurrence and evolution. It reveals the cause, mechanism, location, nature and situation of the disease to varying degrees with certain related pulse symptoms, and provides the basis for prevention and treatment. |
| Priority Review | Review and approval with priority will be implemented for registration applications of new TCMs with clear clinical positioning and obvious clinical value in the following cases:  (1) medicine for the prevention and treatment of major diseases, emerging and unexpected contagious diseases, and rare diseases; (2) medication with an urgent clinical need and a shortage in the market; (3) medication for children; (4) newly discovered raw TCM materials and their preparations, or new of effect parts in raw TCM materials and their preparations; (5) TCMs with a clear profile of medicinal substances and a basically clear mechanism of action. |
| Conditional Approval | In the case that TCMs are used for the treatment of serious life-threatening diseases for which there is no effective treatment method, and TCMs are urgently needed as determined by the State Council's health department or competent department of TCM, if the existing data from clinical trials and empirical evidence based on application experience in human with high quality can show efficacy and predict clinical value, it can be approved with additional conditions, and the relevant matters should be stated in the drug registration certificate. |
| Special Review and Approval Procedure | In the event of a public health emergency, if TCMs are deemed urgently needed by the State Council's health department or competent department of TCM, the TCMs may be directly applied for clinical trials, marketing licenses, or adding functions and indications in accordance with special approval procedures by using empirical evidence based on application experience in human. |
| Rare disease | Drugs listed in the "Catalogue of Rare Diseases" in China. |
| Rare disease drug | Drug used for the prevention, treatment, and diagnosis of rare diseases. |

Abbreviations: TCM, traditional Chinese Medicine.

## Supplementary Table 3. Basic information on new traditional Chinese medicine approved in China.

| Number | Drug name | Approval type | Registration Categories | ATC Classification | Approval Date | Origin |
| --- | --- | --- | --- | --- | --- | --- |
| 1 | Guibai Huayu Capsules | TCM compound preparation | 6 | Genital urinary system and sex hormones | 2013/1/10 | Domestic |
| 2 | Jiuwei Xifeng Granules | TCM compound preparation | 6 | Nervous system | 2013/1/10 | Domestic |
| 3 | Liqi Huoxue Pills | TCM compound preparation | 6.1 | Blood and blood-forming organs | 2013/1/10 | Domestic |
| 4 | Naomaixin Granules | TCM compound preparation | 6.1 | Blood and blood-forming organs | 2013/1/30 | Domestic |
| 5 | Jinxiang Shugan Tablets | TCM compound preparation | 6 | Nervous system | 2013/1/30 | Domestic |
| 6 | Xuangui Dripping Pills | TCM compound preparation | 6 | Genital urinary system and sex hormones | 2013/4/2 | Domestic |
| 7 | Qinsang Jinhai Granules | TCM compound preparation | 6.2 | Dermatological | 2013/4/2 | Domestic |
| 8 | Longxue Tongluo Capsules | TCM single preparation | 5 | Blood and blood-forming organs | 2013/7/3 | Domestic |
| 9 | Hewei Zhixie Capsules | TCM compound preparation | 6.1 | Alimentary tract and metabolism | 2013/8/5 | Domestic |
| 10 | Liuwei Qufeng Huoluo Plaster | TCM compound preparation | 6.2 | Musculo-skeletal system | 2013/8/5 | Domestic |
| 11 | Yuzhi Zhixue Granules | TCM compound preparation | 6.1 | Genital urinary system and sex hormones | 2013/11/4 | Domestic |
| 12 | Yinghua Pills | TCM compound preparation | 6.1 | Genital urinary system and sex hormones | 2013/11/4 | Domestic |
| 13 | Tongxie Soft Capsules | TCM compound preparation | 6 | Alimentary tract and metabolism | 2013/11/4 | Domestic |
| 14 | Tanreqing Capsules | TCM compound preparation | 6.1 | Respiratory system | 2013/12/20 | Domestic |
| 15 | Zhuling Jianpi Capsules | TCM compound preparation | 6 | Alimentary tract and metabolism | 2014/1/16 | Domestic |
| 16 | Qianlie Miniao Suppository | TCM compound preparation | 6.1 | Genital urinary system and sex hormones | 2014/3/4 | Domestic |
| 17 | Ranjiangduoji Capsules | TCM compound preparation | 6.1 | Musculo-skeletal system | 2014/4/9 | Domestic |
| 18 | Sanfeng Tongqiao dropping Pills | TCM compound preparation | 6.2 | Respiratory system | 2014/4/9 | Domestic |
| 19 | Chuanshegan Huangtong Capsules | TCM single preparation | 5 | Respiratory system | 2014/6/17 | Domestic |
| 20 | Zibei Zhike Granules | TCM compound preparation | 6.1 | Respiratory system | 2014/8/11 | Domestic |
| 21 | Sanqi Longxuejie Capsules | TCM compound preparation | 6.2 | Blood and blood-forming organs | 2014/11/6 | Domestic |
| 22 | Xifeng Zhidong Tablets | TCM compound preparation | 6.2 | Nervous system | 2014/11/6 | Domestic |
| 23 | Jin dilian jiedu Tablets | TCM compound preparation | 6 | Respiratory system | 2014/11/21 | Domestic |
| 24 | Mian An Ning Capsules | TCM compound preparation | 6.1 | Nervous system | 2014/12/18 | Domestic |
| 25 | Shezhi Bingfu Ointment | TCM compound preparation | 6 | Dermatological | 2015/1/7 | Domestic |
| 26 | Jianghuang Tongluo capsules | TCM compound preparation | 6 | Cardiovascular system | 2015/2/25 | Domestic |
| 27 | Compound Kushen Colonrelease Capsules | TCM compound preparation | 6.2 | Alimentary tract and metabolism | 2015/2/25 | Domestic |
| 28 | Danlu Capsules | TCM compound preparation | 6.1 | Genital urinary system and sex hormones | 2015/5/4 | Domestic |
| 29 | Shouhui Tongbian Capsules | TCM compound preparation | 6.1 | Alimentary tract and metabolism | 2015/5/20 | Domestic |
| 30 | Tribulus Terrestris Saponin Capsule | TCM single preparation | 5 | Nervous system | 2015/7/8 | Domestic |
| 31 | Huailing Ointment | TCM compound preparation | 6 | Cardiovascular system | 2015/7/8 | Domestic |
| 32 | Jinhua Qinggan Granules | TCM compound preparation | 6.1 | Respiratory system | 2016/9/12 | Domestic |
| 33 | Keyoudu Oiniment | TCM compound preparation | 6 | Dermatological | 2016/10/11 | Domestic |
| 34 | Danlong oral liquid | TCM compound preparation | 6.1 | Respiratory system | 2017/8/29 | Domestic |
| 35 | Guanhuangmu Granules | TCM compound preparation | 6.2 | Genital urinary system and sex hormones | 2018/2/23 | Domestic |
| 36 | Jinrong Granules | TCM compound preparation | 6.1 | Genital urinary system and sex hormones | 2018/12/25 | Domestic |
| 37 | Xiaoer Jingxing Zhike Granules | TCM compound preparation | 6.1 | Respiratory system | 2019/12/18 | Domestic |
| 38 | Shaoma Zhijing Granules | TCM compound preparation | 6.1 | Nervous system | 2019/12/20 | Domestic |
| 39 | Mulberry Twig Alkaloids | TCM single preparation | 5 | Alimentary tract and metabolism | 2020/3/18 | Domestic |
| 40 | Jingu Zhitong Gel | TCM compound preparation | 6.1 | Musculo-skeletal system | 2020/4/13 | Domestic |
| 41 | Lianhua Qingke Tablets | TCM compound preparation | 6.1 | Respiratory system | 2020/5/12 | Domestic |
| 42 | Qingfei Paidu Granules | TCM compound preparation | 3.2 | Respiratory system | 2021/3/2 | Domestic |
| 43 | Huashi Baidu Granules | TCM compound preparation | 3.2 | Respiratory system | 2021/3/2 | Domestic |
| 44 | Xuanfeibaidu Granule | TCM compound preparation | 3.2 | Respiratory system | 2021/3/2 | Domestic |
| 45 | Yishen Yangxin Anshen Tablets | TCM compound preparation | 1.1 | Nervous system | 2021/9/1 | Domestic |
| 46 | Yiqi Tongqiao Pills | TCM compound preparation | 1.1 | Respiratory system | 2021/9/13 | Domestic |
| 47 | Yinqiao Baidu Tablets | TCM compound preparation | 1.1 | Respiratory system | 2021/11/9 | Domestic |
| 48 | Kunyining Granules | TCM compound preparation | 1.1 | Genital urinary system and sex hormones | 2021/11/24 | Domestic |
| 49 | Qizhi Yishen Capsule | TCM compound preparation | 1.1 | Alimentary tract and metabolism | 2021/11/24 | Domestic |
| 50 | Xuanqi Jiangu Pill | TCM compound preparation | 1.1 | Musculo-skeletal system | 2021/11/24 | Domestic |
| 51 | Jieyu Chufan Capsule | TCM compound preparation | 1.1 | Nervous system | 2021/12/14 | Domestic |
| 52 | Huzhen QingFeng Capsule | TCM compound preparation | 1.1 | Musculo-skeletal system | 2021/12/14 | Domestic |
| 53 | Qirui Weishu Capsule | TCM compound preparation | 1.1 | Alimentary tract and metabolism | 2021/12/31 | Domestic |
| 54 | lcaritin | TCM single preparation | 1.2 | Antineoplastic and immunomodulating agents | 2022/1/10 | Domestic |
| 55 | Herba Desmodii Styracifolii Flavonoids Capsules | TCM single preparation | 1.2 | Genital urinary system and sex hormones | 2022/9/14 | Domestic |
| 56 | Adonis Amurensis Oral Ulcer Patch | TCM single preparation | 1.2 | Alimentary tract and metabolism | 2022/12/21 | Domestic |
| 57 | Qijiao Tiaojing Granules | TCM compound preparation | 6.1 | Genital urinary system and sex hormones | 2022/12/27 | Domestic |
| 58 | Linggui Shugan Granules | TCM compound preparation | 3.1 | Respiratory system | 2022/12/27 | Domestic |
| 59 | Shenge Bushen Capsules | TCM compound preparation | 1.1 | Nervous system | 2022/12/29 | Domestic |
| 60 | Sanhan Huashi Granules | TCM compound preparation | 3.2 | Respiratory system | NA | Domestic |
| 61 | Shenyu Ningshen Tablets | TCM compound preparation | 1.1 | Nervous system | 2023/6/8 | Domestic |
| 62 | Pipa Qingfei Granules | TCM compound preparation | 3.1 | Respiratory system | 2023/7/26 | Domestic |
| 63 | Tongluo Mingmu Capsules | TCM compound preparation | 1.1 | Sensory organs | 2023/10/19 | Domestic |
| 64 | Xiaoer Zibei Xuanfei Syrup | TCM compound preparation | 1.1 | Respiratory system | 2023/10/19 | Domestic |
| 65 | Citrus aurantium total flavonoid Tablets | TCM single preparation | 1.2 | Alimentary tract and metabolism | 2023/10/19 | Domestic |
| 66 | Centella Asiatica Cream | TCM single preparation | 1.1 | Dermatological | 2023/11/9 | Imported |
| 67 | Xiaoer Chiqiao Qingre Syrup | TCM compound preparation | 2.2 | Respiratory system | 2023/11/17 | Domestic |
| 68 | Yiguan Jian Granules | TCM compound preparation | 3.1 | Alimentary tract and metabolism | 2023/12/26 | Domestic |
| 69 | Jichuan Jian Granules | TCM compound preparation | 3.1 | Nervous system | 2023/12/26 | Domestic |
| 70 | Shaoyao Gancao Granules | TCM compound preparation | 3.1 | Nervous system | 2024/1/8 | Domestic |
| 71 | Ercha Shangqing Pills | TCM compound preparation | 1.1 | Alimentary tract and metabolism | 2024/1/8 | Domestic |
| 72 | Jiuwei Zhike Oral Liquid | TCM compound preparation | 1.1 | Respiratory system | 2024/2/20 | Domestic |
| 73 | Qinwei Granules | TCM compound preparation | 1.1 | Musculo-skeletal system | 2024/3/12 | Domestic |
| 74 | Wenjing decoction Granules | TCM compound preparation | 3.1 | Genital urinary system and sex hormones | 2024/5/15 | Domestic |
| 75 | Danggui Buxue Decoction Granules | TCM compound preparation | 3.1 | Genital urinary system and sex hormones | 2024/8/20 | Domestic |
| 76 | Wenyang Jiedu Granules | TCM compound preparation | 3.2 | Respiratory system | 2024/8/21 | Domestic |
| 77 | Erdong Decoction Granules | TCM compound preparation | 3.1 | Alimentary tract and metabolism | 2024/11/5 | Domestic |

Abbreviations: TCM, traditional Chinese Medicine. ATC, Anatomical Therapeutic Chemical.

## Supplementary Table 4. New traditional Chinese medicines receiving accelerated procedures from 2013 to 2024.

| Number | Drug Name | Years | Indications | Approval path | Reasons |
| --- | --- | --- | --- | --- | --- |
| 1 | Shaoma Zhijing Granules | 2019 | Tic Disorders (TD) | PR | Innovative medicines with clear therapeutic advantages and medicines for children |
| 2 | Mulberry Twig Alkaloids Tablets | 2020 | Type 2 Diabetes Mellitus | PR | First Innovative Anti-Diabetic Chinese Medicine |
| 3 | Qingfei Paidu Granules | 2021 | COVID-19 | SRAP | Preventive and curative medicines for public emergency response |
| 4 | Huashi Baidu Granules | 2021 | COVID-19 | SRAP | Preventive and curative medicines for public emergency response |
| 5 | Xuanfei Baidu Granules | 2021 | COVID-19 | SRAP | Preventive and curative medicines for public emergency response |
| 6 | Epimedium Soft Capsules | 2022 | Hepatocellular carcinoma | CA and PR | New treatment option for patients with hepatocellular carcinoma |
| 7 | Centella Asiatica Cream | 2023 | Diabetic Foot Ulcers | CA | New treatment option for Wagner grade 1 diabetic foot patients |

Abbreviations: PR, Priority Review; SRAP, Special Review and Approval Procedure; CA, Conditional Approval; COVID-19, Corona Virus Disease 2019.

## Supplementary Table 5. Clinical waiver and new TCM based on real-world study.

| Number | Drug name | Source of formulas | Clinical trial Phase |
| --- | --- | --- | --- |
| 1 | Qingfei Paidu Granules | Ancient classic formulas | Real word study |
| 2 | Huashi Baidu Granules | Ancient classic formulas | Real word study |
| 3 | Xuanfeibaidu Granule | Ancient classic formulas | Real word study |
| 4 | Linggui Shugan Granules | Ancient classic formulas | Clinical waiver |
| 5 | Sanhan Huashi Granules | Ancient classic formulas | Real word study |
| 6 | Pipa Qingfei Granules | Ancient classic formulas | Clinical waiver |
| 7 | Yiguan Jian Granules | Ancient classic formulas | Clinical waiver |
| 8 | Jichuan Jian Granules | Ancient classic formulas | Clinical waiver |
| 9 | Shaoyao Gancao Granules | Ancient classic formulas | Clinical waiver |
| 10 | Wenjing decoction Granules | Ancient classic formulas | Clinical waiver |
| 11 | Danggui Buxue Decoction Granules | Ancient classic formulas | Clinical waiver |
| 12 | Wenyang Jiedu Granules | Ancient classic formulas | Real word study |
| 13 | Erdong Decoction Granules | Ancient classic formulas | Clinical waiver |

## Supplementary Table 6. Clinical trial phases and pivotal clinical trial design for new TCMs.

| Number | Drug name | Source of formulas | Clinical trial Phase | Clinical Value | Pivotal trial design | Enrollment, No. | Main efficacy indicators |
| --- | --- | --- | --- | --- | --- | --- | --- |
| 1 | Guibai Huayu Capsules | Clinical experience formulas | Phase Ⅱ、Ⅲ | Others | A domestic multicenter, randomized, double-blind, positive drug-controlled phase III clinical study, | 446 | Surrogate indicators |
| 2 | Jiuwei Xifeng Granules | Clinical experience formulas | Phase Ⅱ、Ⅲ | Improvement of the patient’s disease-related body functions or quality of life | A domestic multicenter, randomized, open, placebo drug-controlled and positive drug-controlled phase III clinical study | 600 | PRO |
| 3 | Liqi Huoxue Pills | Ancient classic formulas | Phase Ⅱ、Ⅲ | Delayed disease progression | A domestic multicenter, randomized, double-blind, positive drug-controlled phase III clinical study | 480 | Surrogate indicators |
| 4 | Naomaixin Granules | Ancient classic formulas | Phase Ⅱ、Ⅲ | Improvement of the patient’s disease-related body functions or quality of life | A domestic multicenter, randomized, open, placebo drug-controlled and positive drug-controlled phase III clinical study | 520 | PRO |
| 5 | Jinxiang Shugan Tablets | Ancient classic formulas | Phase Ⅱ、Ⅲ | Improvement of the patient’s disease-related body functions or quality of life | A domestic multicenter, randomized, double-blind, positive drug-controlled phase III clinical study | 502 | PRO |
| 6 | Qinsang Jinhai Granules | Pharmacology-based screening of TCM | Phase Ⅱ、Ⅲ | Recovery from disease | A domestic multicenter, randomized, double-blind, positive drug-controlled phase III clinical study | 466 | Surrogate indicators |
| 7 | Longxue Tongluo Capsules | Pharmacology-based screening of TCM | Phase Ⅰ、Ⅱ、Ⅲ | Improvement of the patient’s disease-related body functions or quality of life | A domestic multicenter, randomized, open, placebo drug-controlled and positive drug-controlled phase III clinical study | 465 | PRO |
| 8 | Hewei Zhixie Capsules | Clinical experience formulas | Phase Ⅱ、Ⅲ | Recovery from disease | A domestic multicenter, randomized, double-blind, positive drug-controlled phase III clinical study | 450 | Endpoint indicators） |
| 9 | Liuwei Qufeng Huoluo Plaster | Pharmacology-based screening of TCM | Phase Ⅱ、Ⅲ | Improvement of the patient’s disease-related body functions or quality of life | A domestic multicenter, randomized, double-blind, placebo-controlled phase III clinical study | 404 | PRO |
| 10 | Yuzhi Zhixue Granules | Clinical experience formulas | Phase Ⅱ、Ⅲ | Recovery from disease | A domestic multicenter, randomized, double-blind, positive drug-controlled phase III clinical study | 468 | Endpoint indicators |
| 11 | Yinghua Pills | Clinical experience formulas | Phase Ⅱ、Ⅲ | Recovery from disease | A domestic multicenter, randomized, double-blind, positive drug-controlled phase III clinical study | 466 | Endpoint indicators |
| 12 | Tanreqing Capsules | Clinical experience formulas | Phase Ⅱ、Ⅲ | Others | A domestic multicenter, randomized, double-blind, positive drug-controlled phase III clinical study | 584 | TCM syndromes |
| 13 | Ranjiang Duoji Capsules | Clinical experience formulas | Phase Ⅱ、Ⅲ | Improvement of the condition or symptoms | A domestic multicenter, randomized, open, placebo drug-controlled and positive drug-controlled phase III clinical study | 478 | Surrogate indicators |
| 14 | Sanfeng Tongqiao Dropping Pills | Clinical experience formulas | Phase Ⅱ、Ⅲ | Improvement of the condition or symptoms | A domestic multicenter, randomized, double-blind, positive drug-controlled phase III clinical study | 480 | Surrogate indicators |
| 15 | Chuanshegan Huangtong Capsules | Pharmacology-based screening of TCM | Phase Ⅰ、Ⅱ、Ⅲ | Improvement of the condition or symptoms | A domestic multicenter, randomized, double-blind, positive drug-controlled phase III clinical study | 480 | Endpoint indicators |
| 16 | Zibei Zhike Granules | Clinical experience formulas | Phase Ⅱ、Ⅲ | Delayed disease progression | A domestic multicenter, randomized, double-blind, positive drug-controlled phase III clinical study | 330 | Surrogate indicators |
| 17 | Xifeng Zhidong Tablets | Pharmacology-based screening of TCM | Phase Ⅱ、Ⅲ | Improvement of the patient’s disease-related body functions or quality of life | A domestic multicenter, randomized, double-blind, positive drug-controlled phase III clinical study | 596 | PRO |
| 18 | Jin dilian Jiedu Tablets | Clinical experience formulas | Phase Ⅱ、Ⅲ | Improvement of the condition or symptoms | A domestic multicenter, randomized, double-blind, positive drug-controlled phase III clinical study | 411 | Surrogate indicators |
| 19 | Shezhi Bingfu Ointment | Clinical experience formulas | Phase Ⅱ、Ⅲ | Improvement of the condition or symptoms | A domestic multicenter, randomized, double-blind, positive drug-controlled phase III clinical study | 264 | Surrogate indicators |
| 20 | Compound Kushen Colonrelease Capsules | Clinical experience formulas | Phase Ⅱ、Ⅲ | Delayed disease progression | A domestic multicenter, randomized, double-blind, positive drug-controlled phase III clinical study | 463 | Surrogate indicators |
| 21 | Danlu Capsules | Clinical experience formulas | Phase Ⅱ、Ⅲ | Improvement of the condition or symptoms | A domestic multicenter, randomized, double-blind, positive drug-controlled phase III clinical study | 342 | Surrogate indicators |
| 22 | Shouhui Tongbian Capsules | Ancient classic formulas | Phase Ⅱ、Ⅲ | Improvement of the condition or symptoms | A domestic multicenter, randomized, open, placebo drug-controlled and positive drug-controlled phase III clinical study | 465 | Surrogate indicators |
| 23 | Tribulus Terrestris Saponin Capsules | Pharmacology-based screening of TCM | Phase Ⅰ、Ⅱ、Ⅲ | Improvement of the patient’s disease-related body functions or quality of life | A domestic multicenter, randomized, open, placebo drug-controlled and positive drug-controlled phase III clinical study | 786 | PRO |
| 24 | Huailing Ointment | Ancient classic formulas | Phase Ⅱ、Ⅲ | Improvement of the condition or symptoms | A domestic multicenter, randomized, double-blind, positive drug-controlled phase III clinical study | 423 | Endpoint indicators |
| 25 | Jinhua Qinggan Granules | Medical institution TCM preparations | Phase Ⅱ、Ⅲ | Others | A domestic multicenter, randomized, double-blind, placebo-controlled phase III clinical study | 480 | TCM syndromes |
| 26 | Danlong Oral Liquid | Medical institution TCM preparations | Phase Ⅱ、Ⅲ | Delayed disease progression | A domestic multicenter, randomized, double-blind, positive drug-controlled phase III clinical study | 473 | Surrogate indicators |
| 27 | Guanhuangmu Granules | Ancient classic formulas | Phase Ⅱ、Ⅲ | Improvement of the patient’s disease-related body functions or quality of life | A domestic multicenter, randomized, double-blind, positive drug-controlled phase III clinical study | 480 | PRO |
| 28 | Jinrong Granules | Clinical experience formulas | Phase Ⅱ、Ⅲ | Improvement of the condition or symptoms | A domestic multicenter, randomized, double-blind, positive drug-controlled phase III clinical study | 478 | Endpoint indicators |
| 29 | Xiaoer Jingxing Zhike Granules | Clinical experience formulas | Phase Ⅱ、Ⅲ | Improvement of the condition or symptoms | A domestic multicenter, randomized, double-blind, placebo-controlled phase III clinical study | 474 | Endpoint indicators |
| 30 | Shaoma Zhijing Granules | Clinical experience formulas | Phase Ⅱ、Ⅲ | Improvement of the patient’s disease-related body functions or quality of life | A domestic multicenter, randomized, open, placebo drug-controlled and positive drug-controlled phase III clinical study | 603 | PRO |
| 31 | Mulberry Twig Alkaloids | Pharmacology-based screening of TCM | Phase Ⅰ、Ⅱ、Ⅲ | Delayed disease progression | A domestic multicenter, randomized, open, placebo drug-controlled and positive drug-controlled phase III clinical study | 800 | Surrogate indicators |
| 32 | Jingu Zhitong Gel | Clinical experience formulas | Phase Ⅱ、Ⅲ | Improvement of the patient’s disease-related body functions or quality of life | A domestic multicenter, randomized, double-blind, placebo-controlled phase III clinical study | 576 | PRO |
| 33 | Lianhua Qingke Tablets | Clinical experience formulas | Phase Ⅱ、Ⅲ | Improvement of the condition or symptoms | A domestic multicenter, randomized, double-blind, placebo-controlled phase III clinical study | 480 | Endpoint indicators |
| 34 | Yishen Yangxin Anshen Tablets | Clinical experience formulas | Phase Ⅱ、Ⅲ | Improvement of the patient’s disease-related body functions or quality of life | A domestic multicenter, randomized, double-blind, placebo-controlled phase III clinical study | 480 | PRO |
| 35 | Yiqi Tongqiao Pills | Clinical experience formulas | Phase Ⅱ、Ⅲ | Improvement of the patient’s disease-related body functions or quality of life | A domestic multicenter, randomized, double-blind, placebo-controlled phase III clinical study | 480 | PRO |
| 36 | Yinqiao Baidu Tablets | Clinical experience formulas | Phase Ⅱ、Ⅲ | Recovery from disease | A domestic multicenter, randomized, open, placebo drug-controlled and positive drug-controlled phase III clinical study | 598 | Endpoint indicators |
| 37 | Kunyining Granules | Clinical experience formulas | Phase Ⅱ、Ⅲ | Improvement of the patient’s disease-related body functions or quality of life | A domestic multicenter, randomized, double-blind, placebo-controlled phase III clinical study | 472 | PRO |
| 38 | Qizhi Yishen Capsules | Clinical experience formulas | Phase Ⅱ、Ⅲ | Delayed disease progression | A domestic multicenter, randomized, double-blind, placebo-controlled phase III clinical study | 478 | Surrogate indicators |
| 39 | Xuanqi Jiangu Pills | Clinical experience formulas | Phase Ⅱ、Ⅲ | Improvement of the patient’s disease-related body functions or quality of life | A domestic multicenter, randomized, double-blind, placebo-controlled phase III clinical study | 476 | PRO |
| 40 | Jieyu Chufan Capsules | Clinical experience formulas | Phase Ⅱ、Ⅲ | Improvement of the patient’s disease-related body functions or quality of life | A domestic multicenter, randomized, double-blind placebo drug-controlled and positive drug-controlled phase III clinical study | 560 | PRO |
| 41 | Huzhen QingFeng Capsules | Clinical experience formulas | Phase Ⅱ、Ⅲ | Improvement of the condition or symptoms | A domestic multicenter, randomized, double-blind, placebo-controlled phase III clinical study | 445 | PRO |
| 42 | Qirui Weishu Capsules | Medical institution TCM preparations | Phase Ⅱ、Ⅲ | Improvement of the condition or symptoms | A domestic multicenter, randomized, double-blind, positive drug-controlled phase III clinical study | 480 | Endpoint indicators |
| 43 | Icaritin | Pharmacology-based screening of TCM | Phase Ⅰ、Ⅱ、Ⅲ | Delayed disease progression | A domestic multicenter, randomized, double-blind, positive drug-controlled phase III clinical study | 282 | Endpoint indicators |
| 44 | Herba Desmodii Styracifolii Flavonoids Capsules | Pharmacology-based screening of TCM | Phase Ⅰ、Ⅱ、Ⅲ | Recovery from disease | A domestic multicenter, randomized, double-blind, placebo-controlled phase III clinical study | 605 | Surrogate indicators |
| 45 | Adonis Amurensis Oral Ulcer Patch | Pharmacology-based screening of TCM | Phase Ⅰ、Ⅱ、Ⅲ | Recovery from disease | A domestic multicenter, randomized, double-blind, placebo-controlled phase III clinical study | 480 | Endpoint indicators |
| 46 | Qijiao Tiaojing Granules | Clinical experience formulas | Phase Ⅱ、Ⅲ | Delayed disease progression | A domestic multicenter, randomized, double-blind, positive drug-controlled phase III clinical study | 432 | Endpoint indicators |
| 47 | Shenge Bushen Capsules | Pharmacology-based screening of TCM | Phase Ⅰ、Ⅱ、Ⅲ | Improvement of the patient’s disease-related body functions or quality of life | A domestic multicenter, randomized, open, placebo drug-controlled and positive drug-controlled phase III clinical study | 601 | PRO |
| 48 | Shenyu Ningshen Tablets | Clinical experience formulas | Phase Ⅱ、Ⅲ | Improvement of the patient’s disease-related body functions or quality of life | A domestic multicenter, randomized, double-blind, placebo-controlled phase III clinical study | 479 | PRO |
| 49 | Tongluo Mingmu Capsules | Clinical experience formulas | Phase Ⅱ、Ⅲ | Delayed disease progression | A domestic multicenter, randomized, double-blind, positive drug-controlled phase III clinical study | 416 | Surrogate indicators |
| 50 | Xiaoer Zibei Xuanfei Syrup | Clinical experience formulas | Phase Ⅱ、Ⅲ | Improvement of the condition or symptoms | A domestic multicenter, randomized, double-blind, placebo-controlled phase III clinical study | 453 | PRO |
| 51 | Citrus Aurantium Total Flavonoid Tablets | Pharmacology-based screening of TCM | Phase Ⅰ、Ⅱ、Ⅲ | Recovery from disease | A domestic multicenter, randomized, double-blind, placebo-controlled phase III clinical study | 398 | Endpoint indicators |
| 52 | Centella Asiatica Cream | Pharmacology-based screening of TCM | Phase Ⅲ | Recovery from disease | An international multicenter, randomized, open-label, placebo-controlled phase III clinical study | 173 | Endpoint indicators |
| 53 | Xiaoer Chiqiao Qingre Syrup | Clinical experience formulas | Phase Ⅲ | Improvement of the condition or symptoms | A domestic multicenter, randomized, double-blind, positive drug-controlled phase III clinical study | 295 | Surrogate indicators |
| 54 | Ercha Shangqing Pills | Clinical experience formulas | Phase Ⅱ、Ⅲ | Recovery from disease | A domestic multicenter, randomized, double-blind, placebo-controlled phase III clinical study | 480 | Endpoint indicators |
| 55 | Jiuwei Zhike Oral Liquid | Clinical experience formulas | Phase Ⅱ、Ⅲ | Recovery from disease | A domestic multicenter, randomized, open, placebo drug-controlled and positive drug-controlled phase III clinical study | 630 | Endpoint indicators |
| 56 | Qinwei Granules | Clinical experience formulas | Phase Ⅱ、Ⅲ | Improvement of the condition or symptoms | A domestic multicenter, randomized, double-blind, placebo-controlled phase III clinical study | 476 | Endpoint indicators |

Abbreviations: TCM, traditional Chinese medicine; PRO, Patient-Reported Outcome.

## Supplementary Table 7. New TCMs marketed through the real-world study.

| Drug Name | Trials Design | Trials content |
| --- | --- | --- |
| Qingfei Paidu Granules | A retrospective observational study | A retrospective observational study of Qingfei Paidu granules (treatment for COVID-19), with 3715 cases from more than 60 medical institutions in 28 provinces, from human use empirical evidence to support NDA approval. |
| Huashi Baidu Granules | A retrospective observational study+ PCT | Observational studies (including retrospective and prospective studies) were utilized to initially explore the clinical efficacy and safety of Huashi Baidu Granules. Thereafter, PCTs were conducted to confirm their efficacy further, thereby providing empirical evidence for human use to support the marketing of Huashi Baidu Granules. |
| Xuanfei Baidu Granules | A retrospective observational study | Mining and analyzing real-world data and transforming it into evidence of human application experience that meets the requirements for new TCM submissions. |
| Sanhan Huashi Granules | A retrospective observational study+ PCT | An observational study (retrospective cohort study) was conducted to initially explore the clinical efficacy and safety of Sanhan Huashi Granules and then to validate its effectiveness through a randomized, controlled, open clinical study to provide evidence to support the marketing of Sanhan Huashi Granules. |
| Wenyang Jiedu Granules | A retrospective observational study | Mining and analyzing real-world data and transforming it into evidence of human application experience that meets the requirements for new TCM submissions. |

Abbreviations: COVID-19, Corona Virus Disease 2019; PCT, Pragmatic Clinical Trial; TCM, traditional Chinese medicine.

## Supplementary Table 8. Timing of new TCMs approved for marketing from 2013 to 2024.

| Number | Drug name | IND approval date | NDA submission date | Market authorization date |
| --- | --- | --- | --- | --- |
| 1 | Guibai Huayu Capsule | 2004/8/20 | 2009/3/24 | 2013/1/10 |
| 2 | Jiuwei Xifeng Granules | 2004/8/1 | 2007/7/26 | 2013/1/10 |
| 3 | Liqi Huoxue Pills | 2003/8/25 | 2008/8/14 | 2013/1/10 |
| 4 | Naomaixin Granules | 2004/8/1 | 2009/12/18 | 2013/1/30 |
| 5 | Jinxiang Shugan Tablets | NA | 2007/12/27 | 2013/1/30 |
| 6 | XuanGui DiWan | 2004/2/3 | 2010/2/21 | 2013/4/2 |
| 7 | Qinsangjinhai Granules | 2001/1/1 | 2008/8/28 | 2013/4/2 |
| 8 | Longxue Tongluo Capsule | 2003/8/1 | 2006/6/14 | 2013/7/3 |
| 9 | Hewei Zhixie Capsule | 2004/1 | 2010/6/4 | 2013/8/5 |
| 10 | Liuwei Qufeng Huoluo Plaster | 2005/2/23 | 2009/1/15 | 2013/8/5 |
| 11 | Yuzhi Zhixue Granules | 2004/4/4 | 2011/6/22 | 2013/11/4 |
| 12 | Yinghua Pills | NA | 2011/7/28 | 2013/11/4 |
| 13 | Tongxie Soft Capsule | NA | 2010/4/15 | 2013/11/4 |
| 14 | Tanreqing Capsules | 2003/12/9 | 2011/3/14 | 2013/12/20 |
| 15 | Zhuling Jianpi Capsules | NA | 2006/3/15 | 2014/1/16 |
| 16 | Qianlie Miniao Suppository | 2003/12/29 | 2010/2/22 | 2014/3/4 |
| 17 | Ranjiangduoji Capsules | 2001/5/21 | 2009/12/4 | 2014/4/9 |
| 18 | Sanfeng Tongqiao Dropping Pills | 2004/8/11 | 2010/7/14 | 2014/4/9 |
| 19 | Chuanshegan Huangtong Capsules | 2004/8/1 | 2009/11/10 | 2014/6/17 |
| 20 | ZibeiZhike Granules | 2003/1/1 | 2011/6/9 | 2014/8/11 |
| 21 | Sanqi Longxuejie Capsule | NA | 2009/6/12 | 2014/11/6 |
| 22 | Xifeng Zhidong Tablets | 2004/11/26 | 2009/4/28 | 2014/11/6 |
| 23 | Jin dilian jiedu Tablets | 2002/1/1 | 2006/8/20 | 2014/11/21 |
| 24 | Mian An Ning Capsules | 2005/1/10 | 2009/12/7 | 2014/12/18 |
| 25 | Shezhi Bingfu Ointment | NA | 2012/1/4 | 2015/1/7 |
| 26 | Jianghuang Tongluo Capsules | 2003/4/1 | 2007/12/25 | 2015/2/25 |
| 27 | Compound Kushen Colonrelease Capsules | 2005/1/1 | 2010/7/14 | 2015/2/25 |
| 28 | Danlu Capsules | 2003/12/29 | 2010/1/15 | 2015/5/4 |
| 29 | Shouhui Tongbian Capsules | 2006/1/26 | 2011/10/13 | 2015/5/20 |
| 30 | Tribulus Terrestris Saponin Capsules | 2003/7/1 | 2005/8/24 | 2015/7/8 |
| 31 | Huailing Ointment | 2004/9/20 | 2009/11/2 | 2015/7/8 |
| 32 | Jinhua Qinggan Granules | 2010/12/7 | 2011/12/13 | 2016/9/12 |
| 33 | Keyoudu Oiniment | 2004/3/26 | 2006/11/22 | 2016/10/11 |
| 34 | Danlong Oral Liquid | 2000/1/1 | 2007/10/15 | 2017/8/29 |
| 35 | Guanhuangmu Granules | 2004/1/1 | 2013/5/10 | 2018/2/23 |
| 36 | Jinrong Granules | 2005/4/1 | 2013/7/11 | 2018/12/25 |
| 37 | Xiaoer Jingxing Zhike Granules | 2008/12/26 | 2013/5/28 | 2019/12/18 |
| 38 | Shaoma Zhijing Granules | 2003/8/1 | 2018/1/18 | 2019/12/20 |
| 39 | Mulberry Twig Alkaloids | 2008/9/19 | 2018/11/16 | 2020/3/18 |
| 40 | Jingu Zhitong Gel | 2009/1/1 | 2019/4/4 | 2020/4/13 |
| 41 | Lianhua Qingke Tablets | 2010/1/1 | 2019/9/18 | 2020/5/12 |
| 42 | Yishen Yangxin Anshen Tablets | 2009/5/5 | 2020/6/9 | 2021/9/1 |
| 43 | Yiqi Tongqiao Pills | 2009/3/24 | 2018/4/13 | 2021/9/13 |
| 44 | Yinqiao Baidu Tablets | 2010/1/1 | 2020/11/10 | 2021/11/9 |
| 45 | Kunyining Granules | 2007/12/18 | 2021/1/1 | 2021/11/24 |
| 46 | Qizhi Yishen Capsule | 2008/12/26 | 2020/12/24 | 2021/11/24 |
| 47 | Xuanqi Jiangu Pills | 2009/2/3 | 2020/11/30 | 2021/11/24 |
| 48 | Jieyu Chufan Capsules | 2008/5/8 | 2021/3/10 | 2021/12/14 |
| 49 | Huzhen QingFeng Capsules | 2008/1/1 | 2020/11/28 | 2021/12/14 |
| 50 | Qirui Weishu Capsules | 2001/1/1 | 2020/12/29 | 2021/12/31 |
| 51 | lcaritin | 2013/1/25 | 2021/4/10 | 2022/1/10 |
| 52 | Herba Desmodii Styracifolii Flavonoids Capsules | 2007/11/26 | 2021/7/2 | 2022/9/14 |
| 53 | Adonis Amurensis Oral Ulcer Patch | 2008/10/15 | 2018/3/28 | 2022/12/21 |
| 54 | Qijiao Tiaojing Granules | 2001/1/1 | 2012/10/31 | 2022/12/27 |
| 55 | Linggui Shugan Granules | - | 2022/10/1 | 2022/12/27 |
| 56 | Shenge Bushen Capsules | 2005/5/27 | 2022/1/7 | 2022/12/29 |
| 57 | Shenyu Ningshen Tablets | 2005/8/19 | 2022/5/5 | 2023/6/8 |
| 58 | Pipa Qingfei Granules | - | 2023/1/28 | 2023/7/26 |
| 59 | Tongluo Mingmu Capsules | 2005/11/7 | 2022/9/2 | 2023/10/19 |
| 60 | Xiaoer Zibei Xuanfei Syrup | 2010/1/1 | 2022/9/29 | 2023/10/19 |
| 61 | Citrus Aurantium Total Flavonoid Tablets | 2008/8/19 | 2022/9/21 | 2023/10/19 |
| 62 | Centella Asiatica Cream | 2017/1/1 | 2021/3/12 | 2023/11/9 |
| 63 | Xiaoer douqiao Qingre Syrup | 2018/5/17 | 2023/1/28 | 2023/11/17 |
| 64 | Yiguan Jian Granules | - | 2023/7/18 | 2023/12/26 |
| 65 | Jichuan Jian Granules | - | 2023/5/25 | 2023/12/26 |
| 66 | Shaoyao Gancao Granules | - | 2023/7/25 | 2024/1/8 |
| 67 | Ercha Shangqing Pills | 2011/4/6 | 2023/2/9 | 2024/1/8 |
| 68 | Jiuwei Zhike Oral Liquid | 2010/9/1 | 2022/11/11 | 2024/2/20 |
| 69 | Qinwei Granules | 2009/3/24 | 2022/10/10 | 2024/3/12 |
| 70 | Wenjing decoction Granules | - | 2023/6/28 | 2024/5/15 |
| 71 | Danggui Buxue Decoction Granules | - | 2023/12/12 | 2024/8/20 |
| 72 | Wenyang Jiedu Granules | - | 2023/10/14 | 2024/8/21 |
| 73 | Erdong Decoction Granules | - | 2024/1/12 | 2024/11/5 |

Abbreviations: IND, Investigational New Drug; NDA, New Drug Application; NA, not available. The short horizontal line (-) indicates that the drug has not been submitted to the Investigational New Drug Application (IND) due to the application of the clinical trial exemption clause, so there is no record of the relevant submission date.
